# Alginate-based hydrogels as drug delivery vehicles in cancer treatment and their applications in wound dressing and 3D bioprinting

**DOI:** 10.1186/s13036-020-0227-7

**Published:** 2020-03-13

**Authors:** Farhad Abasalizadeh, Sevil Vaghefi Moghaddam, Effat Alizadeh, Elahe akbari, Elmira Kashani, Seyyed Mohammad Bagher Fazljou, Mohammadali Torbati, Abolfazl Akbarzadeh

**Affiliations:** 1 Department of Traditional Medicine, Faculty of Traditional Medicine, Tabriz University of Medical Sciences, Tabriz, Iran; 2 Drug Applied Research Center, Tabriz University of Medical Sciences, Tabriz, Iran; 3 Department of Medical Biotechnology, Faculty of Advanced Medical Sciences, Tabriz University of Medical Sciences, Tabriz, Iran; 4 Higher Education Institute of Rab-Rashid, Tabriz, Iran; 5 Department of Medical Nanotechnology, Faculty of Advanced Medical Sciences, Tabriz University of Medical Sciences, Tabriz, Iran; 6 Department of Food Science and Technology, Faculty of Nutrition, Tabriz University of Medical Sciences, Tabriz, Iran; 7 Tuberculosis and Lung Disease Research Center of Tabriz, Tabriz University of Medical Sciences, Tabriz, 5154853431 Iran; 8 Universal Scientific Education and Research Network (USERN), Tabriz, Iran

**Keywords:** Alginate hydrogels, Drug delivery, Cancer, Wound dressing, 3D bioprinting

## Abstract

Hydrogels are a three-dimensional and crosslinked network of hydrophilic polymers. They can absorb a large amount of water or biological fluids, which leads to their swelling while maintaining their 3D structure without dissolving (Zhu and Marchant, Expert Rev Med Devices 8:607–626, 2011). Among the numerous polymers which have been utilized for the preparation of the hydrogels, polysaccharides have gained more attention in the area of pharmaceutics; Sodium alginate is a non-toxic, biocompatible, and biodegradable polysaccharide with several unique physicochemical properties for which has used as delivery vehicles for drugs (Kumar Giri et al., Curr Drug Deliv 9:539–555, 2012). Owing to their high-water content and resembling the natural soft tissue, hydrogels were studied a lot as a scaffold. The formation of hydrogels can occur by interactions of the anionic alginates with multivalent inorganic cations through a typical ionotropic gelation method. However, those applications require the control of some properties such as mechanical stiffness, swelling, degradation, cell attachment, and binding or release of bioactive molecules by using the chemical or physical modifications of the alginate hydrogel. In the current review, an overview of alginate hydrogels and their properties will be presented as well as the methods of producing alginate hydrogels. In the next section of the present review paper, the application of the alginate hydrogels will be defined as drug delivery vehicles for chemotherapeutic agents. The recent advances in the application of the alginate-based hydrogels will be describe later as a wound dressing and bioink in 3D bioprinting.

## Introduction

### Hydrogels

Hydrogels are three-dimensional networks in which hydrophilic polymers crosslink together. They could swell by absorbing the large quantities of water or biological fluids while keeping their network structure. These compounds were similar to the living tissue because of their high-water capacity, penetrability, and consistency. Recently, a lot of research was done on the preparation of the transdermal membranes using polysaccharides [1–3]. Among the most widely proposed hydrophilic polymers in hydrogels preparation, polysaccharides had a number of benefits versus the synthetic polymers. Hydrogels had prepared from polysaccharides attracted the attention of researches, due to the applications in biomedical and other areas like those of pharmacy, chemical engineering, agriculture, and food. Despite the limitations of the natural polysaccharides in their reactivity and processability, they could also be used by cross-linking, blending and etc. after modification [4]. Sodium alginate (SA) was one of the most commonly used natural polysaccharides which was obtained from the condensation of β-D-mannuronic acid (M) and 1*–*4 linked *α*-L-guluronic residues (G). SA had the unique property of the gel-formation in the presence of the multivalent cations in aqueous media and broadly used as a gelling agent in the food industry. The gelation and cross-linking of alginate were achieved by the exchange of sodium ions with multivalent cations. The resulted cross-linked hydrogel was useful in the controlled release of the bioactive molecules [5, 6] and tissue engineering as a scaffold.

## Alginate

### Chemical structure

**Fig. 1:**
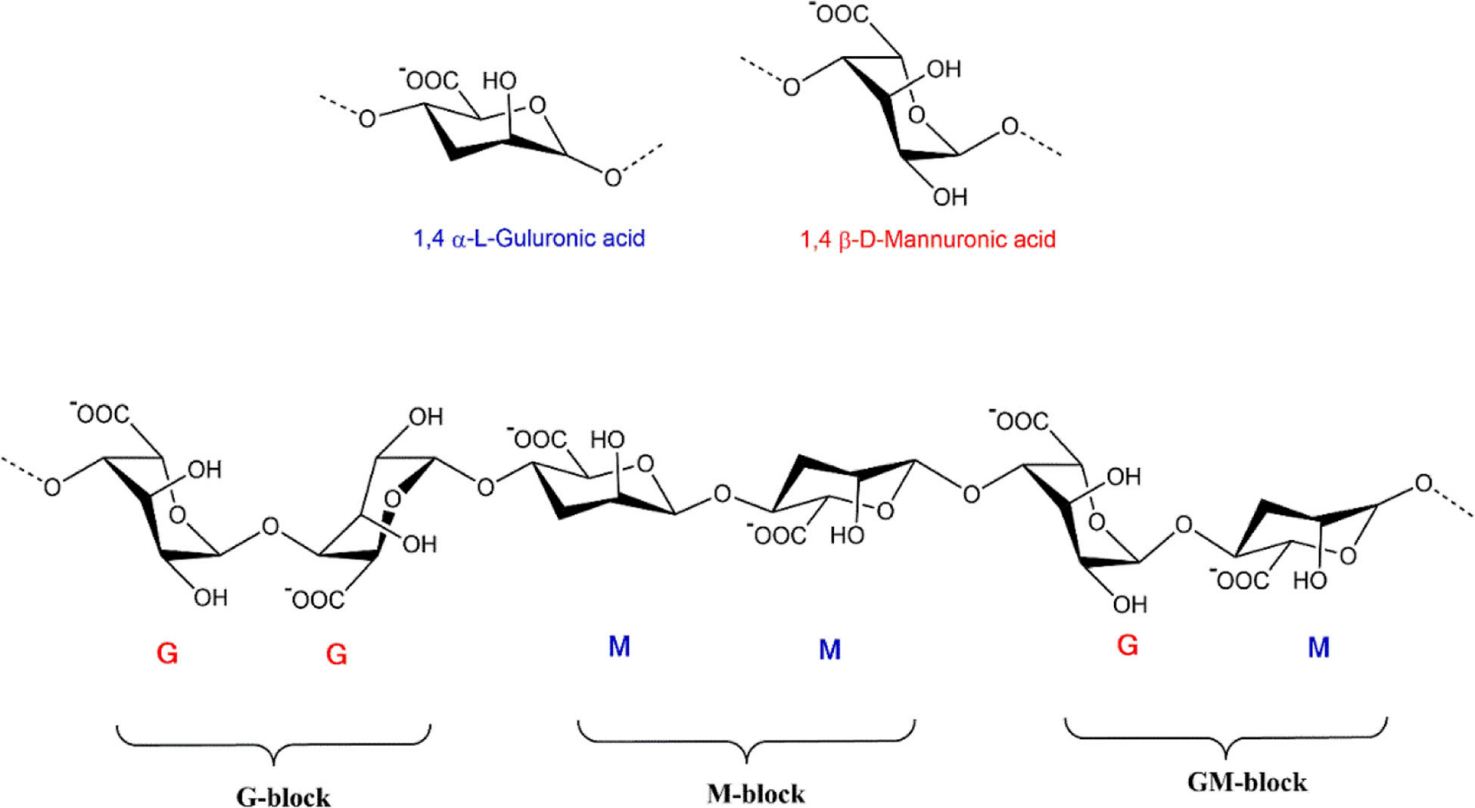
The conformation of monomers and blocks distribution of alginate salt

Alginate (ALG) is composed of the irregular blocks of *β*-D-mannuronic acid (M) and 1*–*4 linked *α*-L-guluronic residues (G) which is the water-soluble linear polysaccharide. Its block-like structure is organized in the pattern of homogenous (poly-G, poly-M) or heterogeneous (MG) pattern [7]. Because of the specific profiles of the monomers and their modes of linkage in the polymer, the geometries of the G-block regions, M-block regions, and alternating regions are considerably diverse [8]. Increasing G blocks in alginate and the molecular weight of the polymer can form stronger or fragile ALG gels. Given the insolubility of alginic acid in water or organic solvent, monovalent alginate salts are soluble and form stable solutions. Decreasing pH below the pKa 3.38–3.65 causes the precipitation of the alginate biopolymer. Ionic strength and gelling ions are the other factors that affect the solubility of the alginate salts (Fig. 1).

### Sources

**Fig. 2:**
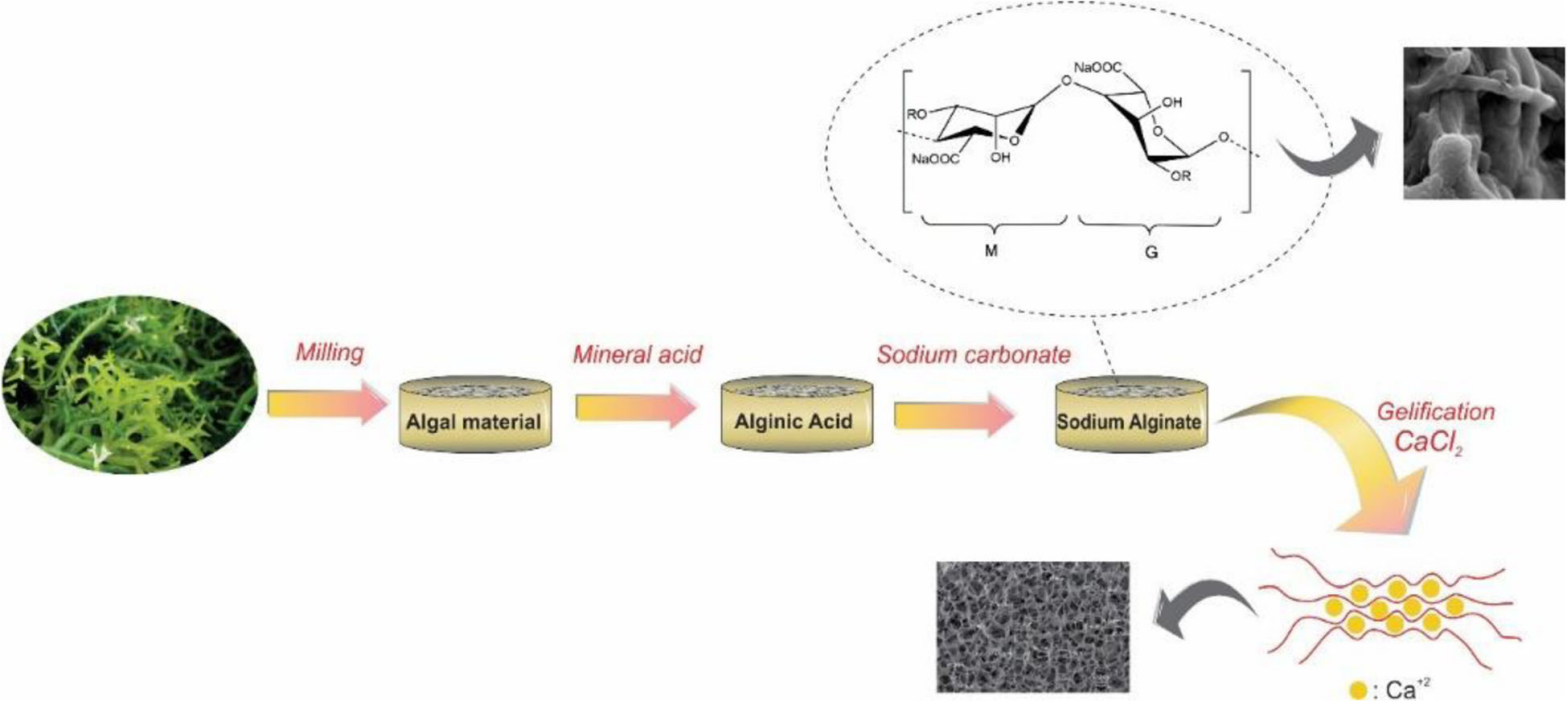
A typical process for the extraction of sodium alginate from brown algae followed by gellification in the presence of CaCl_2_

Alginates are extracted from three species of brown*-*algae-cell walls including *Ascophyllum nodosum*, *Laminaria Hyperborean*, *Macrocystis pyrifera*, and several bacteria (*Azotobacter vinelandii*, *Pseudomonas spp.*), in which alginate involves up to 40% of their dry weights [9]. This term generally applies to all alginic acid derivatives and their salts. Alginic acid is extracted from the algae by using dilute HCl. Then either NaCl or CaCl_2_ is added to the filtrate extract, resulting in the fibrous precipitation of sodium or calcium alginate. Finally, sodium alginate powder is obtained after acidic treatment of the precipitate followed by further purification and lyophilization [10]. One of the main properties of the sodium alginate is the ability to form hydrogel which is mainly because of the substitution of sodium ions of the guluronic acid residues by different divalent cations (Ca^2+^, Sr^2+^, Ba^2+^, and etc.). Thereupon binding of the divalent cation to the α-L-guluronic block (and between two different chains), a 3D network is formed. The process for extracting ALG from seaweeds is plain, which usually begins using dilute mineral acid to treat the dried raw material. The resulting alginic acid is transformed into hydrophilic sodium salt in the presence of sodium carbonate, which can be easily converted to acid or salt (Fig. 2) [11].

**Fig. 3:**
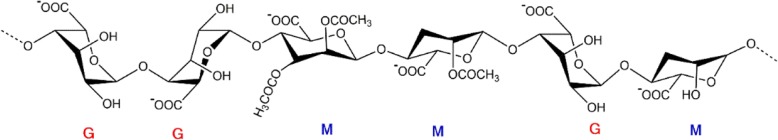
Chemical structure and conformation of guluronic acid (G) and mannuronic acid (M) residue in bacterial alginate

Potentially alginates can be acetylated. Unlike alginates from seaweed (Acetylation = 0), bacterial alginates have a high degree of acetylation (Fig. 3). The approximate amount of M/G ratios and their acetylation varies according to the bacterial species and growth conditions. Acetyl groups affect the viscosity and flexibility of the alginate as well as M/G ratio and MW [12–14].

### General properties of ALG

ALG is commercially accessible in disparate composition, molecular weight, and distribution patterns of M-block and G-block, which are the causes of their physicochemical properties, such as viscosity, the transformation of the sol /gel and water absorption. The viscosity of the alginate is dependent on the pH of the solution. Decreasing in the pH value causes the increase in the viscosity due to the protonation of the carboxylate groups in the alginate backbone that leads to form the hydrogen bond. In commercial ALG, the molecular weight which is the average number of the molecules in the sample varies between 33,000 and 400,000 g/mol. Increasing the molecular weight of the alginate can affect the physical properties of the resultant gels (e.g., high molecular weight alginate solution becomes greatly viscous). In contrast to the water solubility of ALG monovalent salts and ALG esters, alginic acid in both water and organic solvents is insoluble [11]. ALG with poly-M or poly-G structures precipitates at low pH, while those with alternative MG-blocks are soluble at the same condition [15]. ALG is used in the food and pharmaceutical industries as suspension and emulsion stabilizers, thickeners, and viscosity-increasing agents because of its exclusive ability of the sol/gel transition that leads to form the semisolid or solid structures [16].

### Gel formation

Alginate gelation is done under the mild conditions using non-toxic reactants. It has capability to form the gels by substitution of the sodium ions from the guluronic acids with the divalent cations such as Ca^+ 2^ which crosslink the polymer chains through the “egg-box” model [17–19] or by decreasing the pH value below the pKa of ALG monomers by using the lactones like d-glucono-훿-lactone [11]. During the studies, it was determined that various factors such as composition, molecular weight as well as the gel-forming kinetics and the cation have a significant influence in several critical properties that involved in porosity, swelling behavior, constancy, biodegradability, gel strength, and the gel’s immunological characteristics and biocompatibility [9].

**Fig. 4:**
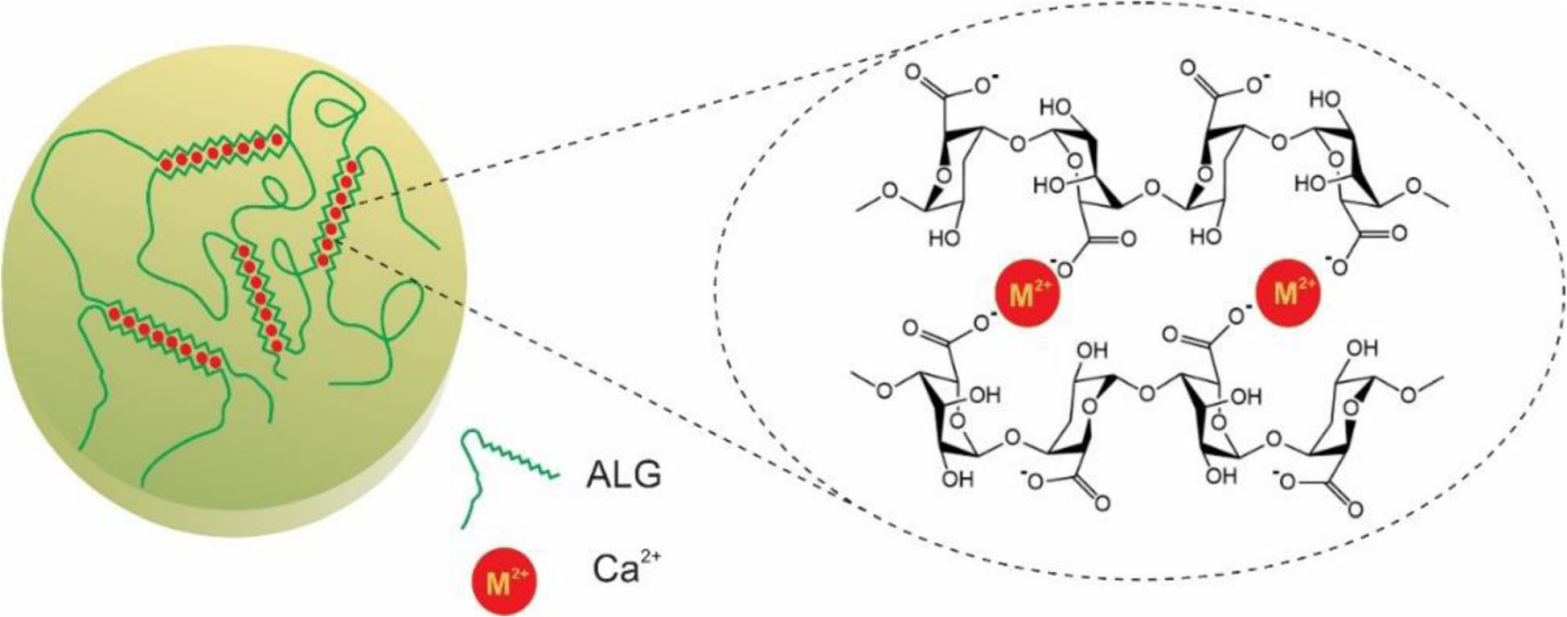
Egg-box structure for alginate gelation as a result of ionic interaction between alginate and a divalent cation

An essential factor in controlling the gelation process is the gelation rate. Slow gelation provides the mechanical integrity for uniforming the gel structures [20]. While calcium cations are responsible for the fast and uncontrollable ALG gelation, carboxylate groups, phosphate groups compete with calcium ions and as a result, the gelation process of the ALG is delayed [21]. It should be mentioned that calcium chloride, the most significant source of the calcium cations, is responsible for the rapid and uncontrollable ALG gelation while low-solubility of the calcium sulfate and calcium carbonate extend the gel formation [22]. Also, the gelation rate depends on the temperature so that reduce temperatures cause a reduction in Ca^2+^ reactivity [23]. The most important factor affected on physicochemical properties of ALG is M/G ratio which alters between different types of brown algae and even various pieces of the same plant. G-blocks are bent or distorted while M-blocks extended ribbon-like form. Only alginate G-blocks are assumed to take part in the intermolecular cross-linking with divalent cations such as Ca^2+^ to produce hydrogels so that if two regains of G-blocks aligned side by side, a diamond shape hole with dimension appropriate to Ca^2+^is formed [24]. The cross-linking procedure is mainly achieved by the replacement of the sodium ions of G-blocks with the divalent cations such as Ca^2+^ and bending of guluronic groups to create the structure like the egg box (Fig. 4) [2]. Therefore, ALG gels with plenty of poly G-block units are known to be fragile, rigid, and mechanically more stable. They also show high porosity, a little amount of shrinkage during the gelation and never swell after drying. When ALG enriched with M-blocks the gels became gradually soft and more elastic with reduced shrinkability and porosity [18]. The shrinkage and flexibility of ALG gel arewere determined by the MG-blocks [25]. Similar to ALG with a large number of residues from G-blocks, ALG with dominant M-block content, exchange ions more easily as a result of high-water absorption [18, 19, 26]. It is noteworthy that the chemical structures of ALG depended on the source of the polymer. Bacterial alginate produced from Azotobacter has a high concentration of the G-blocks and its gels have a relatively high stiffness [27].

### Methods to produce alginate hydrogel

The gelling process is the interconnection of the macromolecular chains together, results in gradually lengthy branched, yet soluble polymer which is called ‘sol’. Progression of the linking procedure could produce an infinite polymer called ‘gel’ or ‘network’ with a giant branched polymer that leads to the reduced solubility. The transition from sol to gel system called ‘sol-gel’ transition or ‘gelation’ [28].

Alginate hydrogels with biomedical applications could be classified into ‘physical’ or ‘reversible’ gels, when some factors, including molecular entanglements, hydrophobic interactions and ionic or hydrogen bonding keep networks together, and ‘chemical’ or ‘permanent’ gels, when stable covalent bonds crosslinked networks together [29]. Many approaches were used to prepare the alginate hydrogels, including ionic crosslinking, covalent crosslinking, phase transition (thermal gelation), Cell crosslinking and free radical polymerization [30, 31].

#### Ionic crosslinking

Typically, the alginate hydrogel in aqueous solution could be produced in the presence of the divalent cations such as Ca^+ 2^, Mg^+ 2^, etc. as the ionic crosslinking agent. It is assumed that they interact with polymer branch G blocks to create the ionic bridges that lead to the so-called egg-box structure. However, M blocks have weak junctions with the divalent cations.

CaCl_2_ is the most commonly exploited ionic crosslinking agent of the alginate hydrogel. Owing to the high solubility of CaCl_2_ in the aqueous medium, the alginate gelation rate is too high to control. Moreover, the gel uniformity and strength are directly affected by the speed of gelation. The decrease in gelation rate produces more uniform structures and greater mechanical integrity.

In order to retarded gelation speed, CaSO_4_ and CaCO_3_ could be used instead. Their poor solubility in aqueous solution increases the aging time of the alginate. Also, a phosphate-containing buffer (e.g., sodium hexametaphosphate) could be used since phosphate groups in the buffer competed with the carboxylate groups of the alginates in the reaction with calcium ions, and lowering the gelation.

Temperature is one of the more important factors that influence the gelation rate and mechanical properties of resultant gel; that is, lowering the temperature reduces the reactivity of the divalent cations that leads to decrease in the gelation rate followed by the high ordered crosslinked network that in turn increase mechanical properties.

#### Covalent crosslinking

Covalent crosslinking covered here involves utilizing a crosslinking agent to junk two polymer chains. The crosslinking of the natural and synthetic polymers could be achieved through the reaction of their functional groups (including -OH, −COOH, and -NH_2_) with crosslinkers such as glutaraldehyde, adipic acid dihydrazide, poly (ethylene glycol)-diamine.

**Fig. 5:**
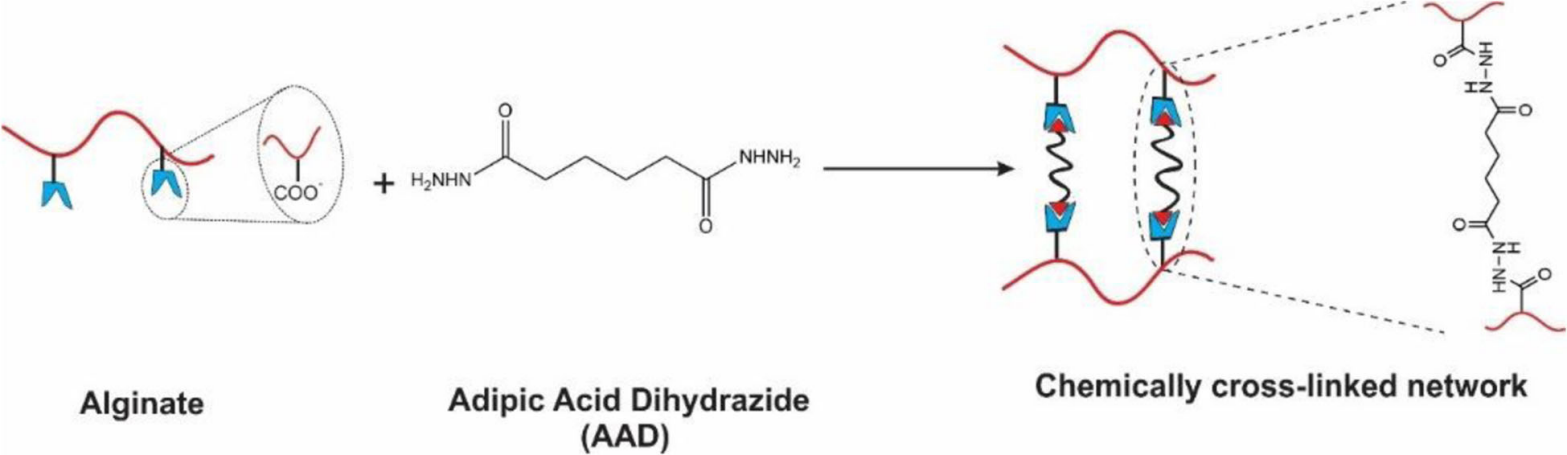
Schematic showing of covalent crosslinking of alginate using adipic acid dihydrazide as cross-linker

As depicted in Fig. 5, the alginate gels with covalent crosslinking are normally generated by the reaction between carboxylic groups in two different alginate branches and a crosslinking molecule possessing primary diamines. The alginate hydrogel’s mechanical properties and swelling degrees could dramatically be affected by variable types of crosslinking molecules and controllable crosslinking density. Crosslinking density directly influences on the mechanical properties of the hydrogels; however, swelling property is significantly controlled by the type of the crosslinking molecules. Utilizing hydrophilic crosslinking molecules as a second macromolecule (e.g., PEG) compensates for the reduction of the hydrophilic character during the crosslinking process. The mentioned approach could candidate them for biomedical as well as other applications in controlling the properties of hydrogels with various combinations of the crosslinking densities and kinds of crosslinking molecules.

#### Phase transition

Another method of producing the hydrogels is the thermoresponsive phase transition that occurs when the temperature rises above the low critical solution temperature (LCST) [31]. poly(N-isoropylacrylamide) (PNIPAM) is an example of a thermosensitive polymer that was most widely investigated [32–34]. PNIPAM hydrogel promotes coil to globule phase transition at lower critical solution temperature (LCST) around 32 °C. Utilizing the temperatures superior the LCST makes PNIPAM precipitate as a solid gel out of a solution. By contrast in the temperatures below the LCST, solid PNIPAM changes to the liquid form. Therefore, the polymer shows a reversible phase transition behavior [35]. Increasing the temperature weakens the intermolecular hydrogen bonds between PNIPAM hydrophilic groups (C=O and N–H) and water molecules that leads to the water release, while strengthens the PNIPAM intramolecular hydrogen bonds, which increase the hydrophobic interaction caused by the isopropyl polymer groups and results in aggregation of PNIPAM into a solid gel (globule phase) [36, 37]. When the temperature comes below LCST, PNIPAM branches unfold and change into the random coils as a result of the re-established hydrogen bonds between water molecules and PNIPAM hydrophilic groups, which tends to be a free-flowing polymer solution [38, 39].

**Fig. 6:**
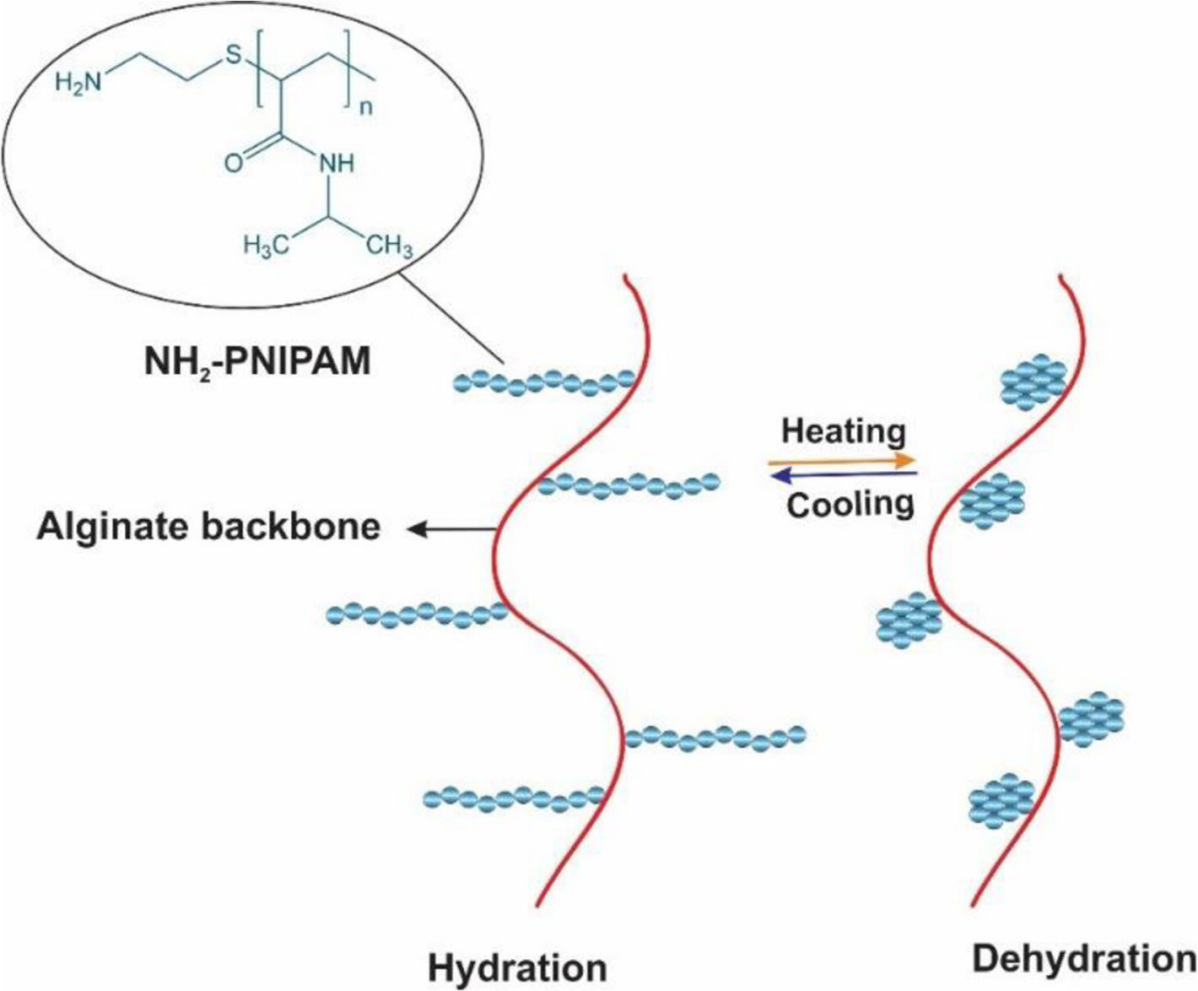
Schematic of the temperature-dependent property of NH_2_-PNIPAM-g-Alg polymer. PNIPAM = poly(N-isopropylacrylamide)

By incorporating PNIPAM into the backbone of alginate, the resultant copolymer could achieve the thermos-responsive nature aside from enhanced mechanical strength and biocompatibility [40, 41]. Therefore, in minimally invasive surgery in order to prevent the postsurgical adhesions which could cause severe clinical complications, PNIPAM based hydrogel solution could be injected under the LCST into the peritoneal cavity that transforms to the gel at body temperature [42]. In comparison to open surgery this procedure is much easier, cost-effective, and time-saving [43]. Figure 6 shows the temperature dependence behavior of PNIPAM-g-alginate hydrogel which has been produced by forming a covalent bond between amino groups of NIPAM copolymer (PNIPAM-NH_2_) and carboxyl groups of the alginates [32]. The proportion of the swelling was particularly affected by the phase transition behavior of the PNIPAM that was attached only on the surface of the pores so that at temperatures above 32 °C the swelling ratio was significantly reducing.

Another way to fabricate the thermos-responsive alginate hydrogel is incorporating with Pluronic F127. In order to overcome the Pluronic F127 drawbacks, which include weak mechanical strength and swift erosion, alginate could physically blend or chemically crosslinked with it [44].

#### Cell crosslinking

**Fig. 7:**
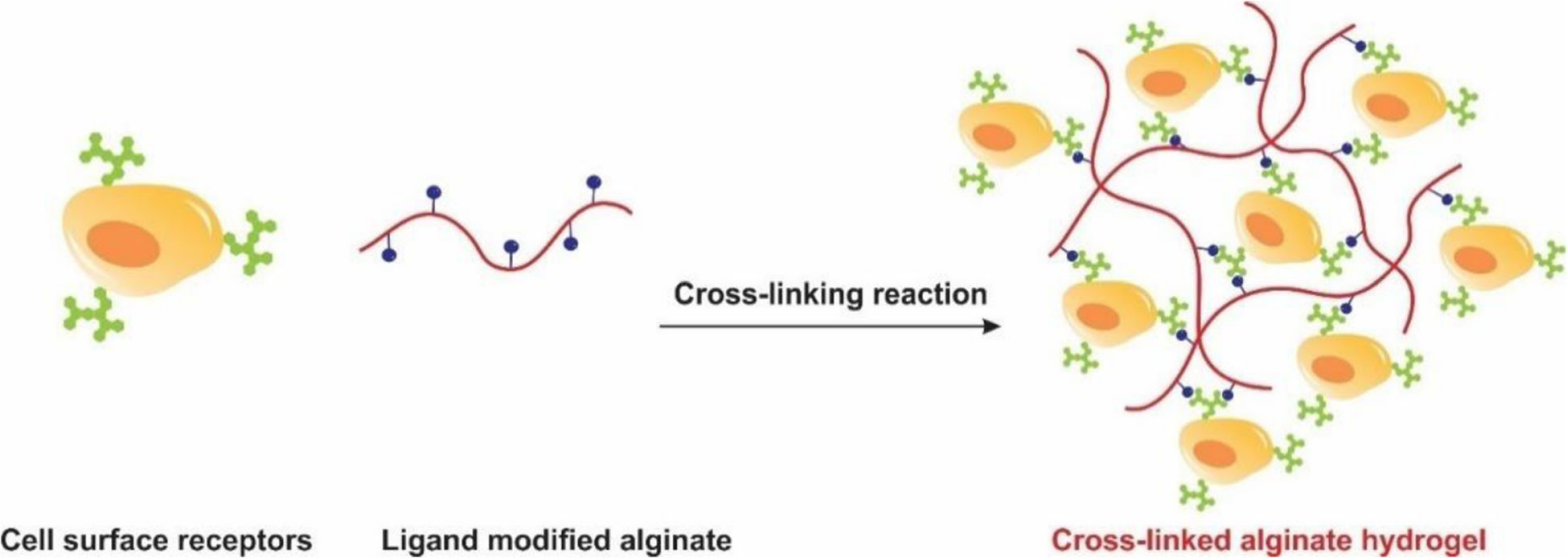
Schematic showing of construction of cell crosslinked hydrogel of ligand modified alginate

Aside from a large number of the physical and chemical approaches to produce the alginate gels, the ability of cells to participate in the gel formation should not be ignored. Cells can crosslink to the polymers and form the gels if the polymer chains have specific ligands to bind to the receptors on the surface of the cells (Fig. 7). In spite of the biocompatibility and high mechanical strength, alginate chains have no bioactive ligands for anchoring to the cells. When alginate chains modified with the cell adhesion peptides like arginine, glycine, aspartic acid sequence (Arg-Gly-Asp, RGD), the ability of cells to bind to the chains, results in long-distance, reversible polymer network even in the absence of a chemical crosslinking agent. The addition of cells to the RGD modified alginate solution produces uniform dispersion of the cells within the solution that leads to the formation of a polymer network through the specific receptor-ligand interactions [45, 46].

#### Free radical polymerization

Free radical polymerization is a method that transforms a linear polymer into a 3D polymer network that could be achieved at the temperature and physiological pH in the presence of some suitable chemical initiators [47, 48]. Modified alginate hydrogels with chains which are appropriate for photocrosslinking have recently received attention for various applications. In this system, alginate chains are modified with functional groups (i.e., methacrylates) followed by the free-radical polymerization in the presence of a photoinitiator and UV light irradiation. Furthermore, cells or bioactive molecules could also encapsulate and crosslink in the physiological conditions during the polymerization [49–51]. In spite of the high cell viability that comes from ionic crosslinking, the resultant hydrogel is not injectable and stable and is more suitable for the clinical use. Free radical polymerization leads to the formation of the covalent crosslinking between methacrylate groups, instead of the ionic crosslink formed by the calcium in the nonmodified alginate [52–54]. In situ crosslinking between the chains is the main privilege of the photocrosslinkable hydrogels. In the treatment of the cartilage defect, the solution of the alginate-cell could be inserted into the cartilage deficiency and crosslinked by the UV light to fill the injury’s irregular shape [55, 56]. That method decreases the need to manufacture the chondrocyte embedded hydrogel in vitro and then use an invasive procedure to implant it into the joint. The ability to match the defect’s form allows the better compatibility between the native tissue and the constructed scaffold. Furthermore, methacrylated alginate hydrogels are capable of controlling the mechanical properties, swelling ratios and degradation levels by altering the surface and photoinitiator concentrations and UV exposure levels [53, 54].

**Fig. 8:**
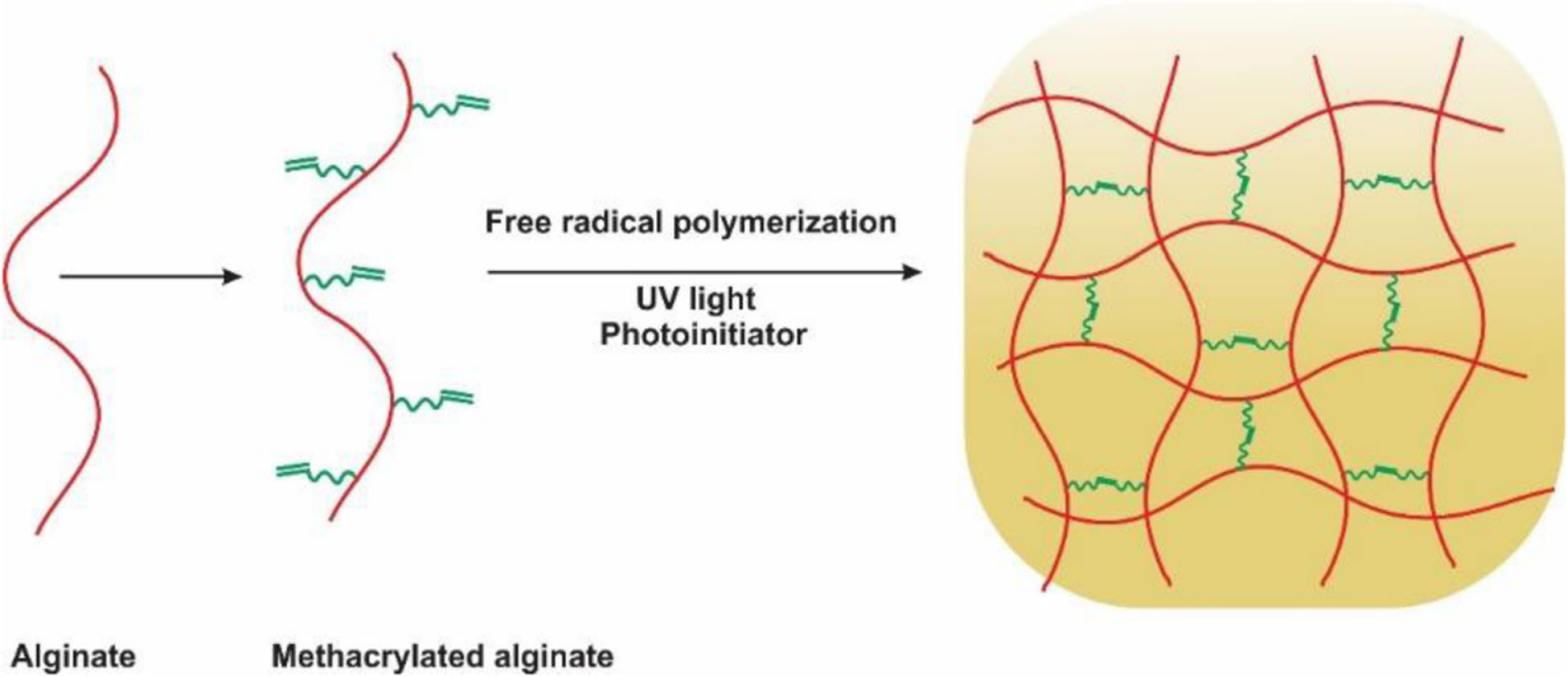
Schematic representation of photocrosslinking of methacrylated alginate

The special benefit of the chain-growth polymerization is the simplicity which a number of chemicals could be integrated into the hydrogel by simply combining and copolymerizing the derived macromeres of choice [48, 57, 58]. That polymerization reaction induces a fluid-solid phase transformation under the physiologic conditions and is ideal for the encapsulation of the cells in situ (Fig. 8).

#### Click chemistry

**Fig. 9:**
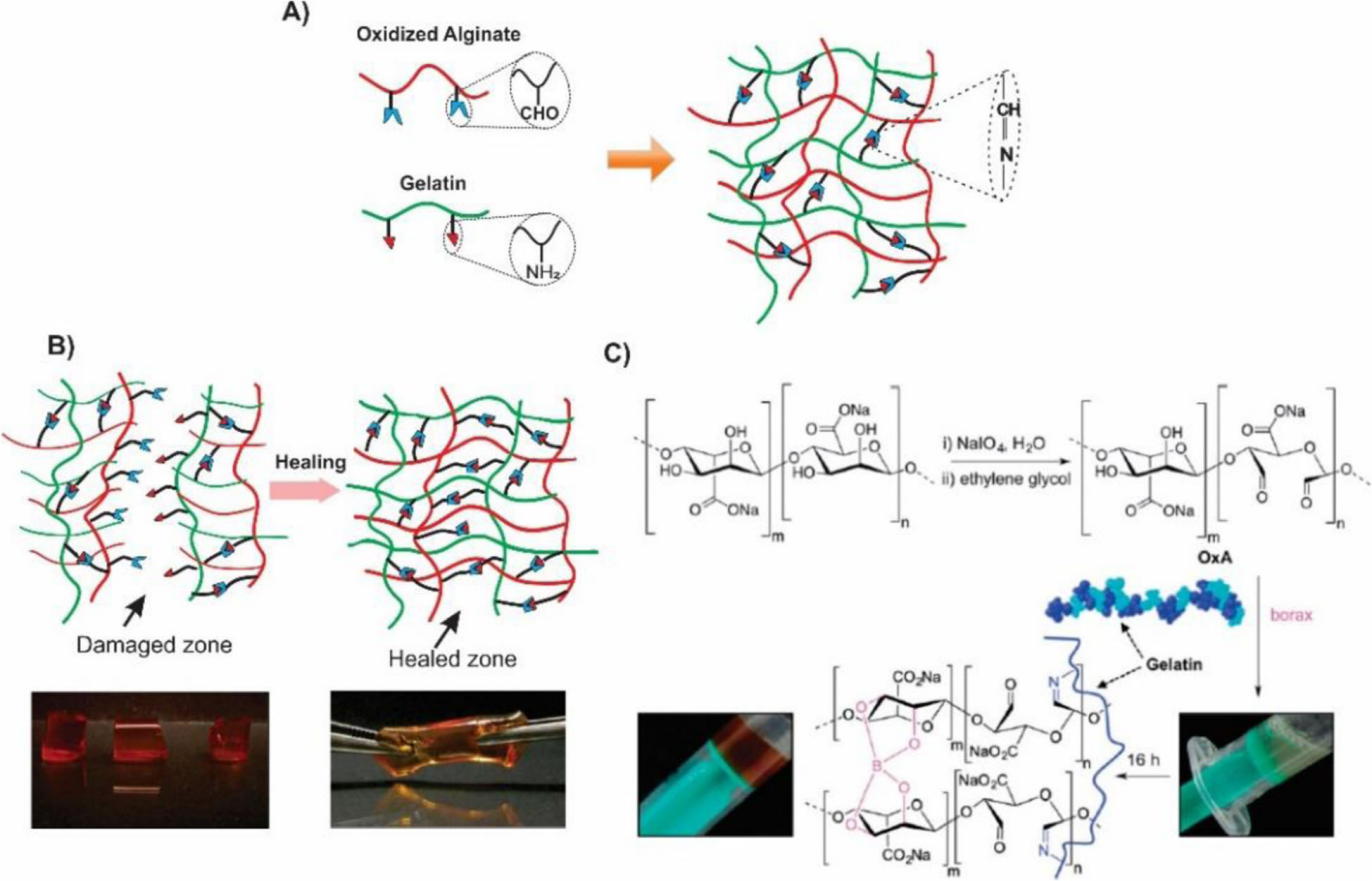
Release and cytotoxicity assessment of EPI using EPI & AG-G5 nanogels as a pH-responsive carrier. **a** Schematic representation of EPI & AG-G5 nanogels synthesis. **b** Time-dependent cumulative release profiles of EPI from nanogels at two pH 7.4 and 5.5. **c** viability assessment of MCF-7 cells after 48 h incubation with the appropriate amount of nanogels equivalent to EPI concentrations. Statistical significance was carried out by Two-way ANOVA with Tukey’s multiple comparisons test between groups using GraphPad Prism 6.0 software. Statistically significant values were denoted by * (*p* < 0.05), ** (*p* < 0.005), and *** (*p* < 0.001). Statistically insignificant values were represented by ‘ns’ [101]. Matai, I. and P. Gopinath, Chemically cross-linked hybrid nanogels of alginate and PAMAM dendrimers as efficient anticancer drug delivery vehicles. ACS Biomaterials Science & Engineering. 2016, 2(2):213–223. Copyright (2020)

**Fig. 10:**
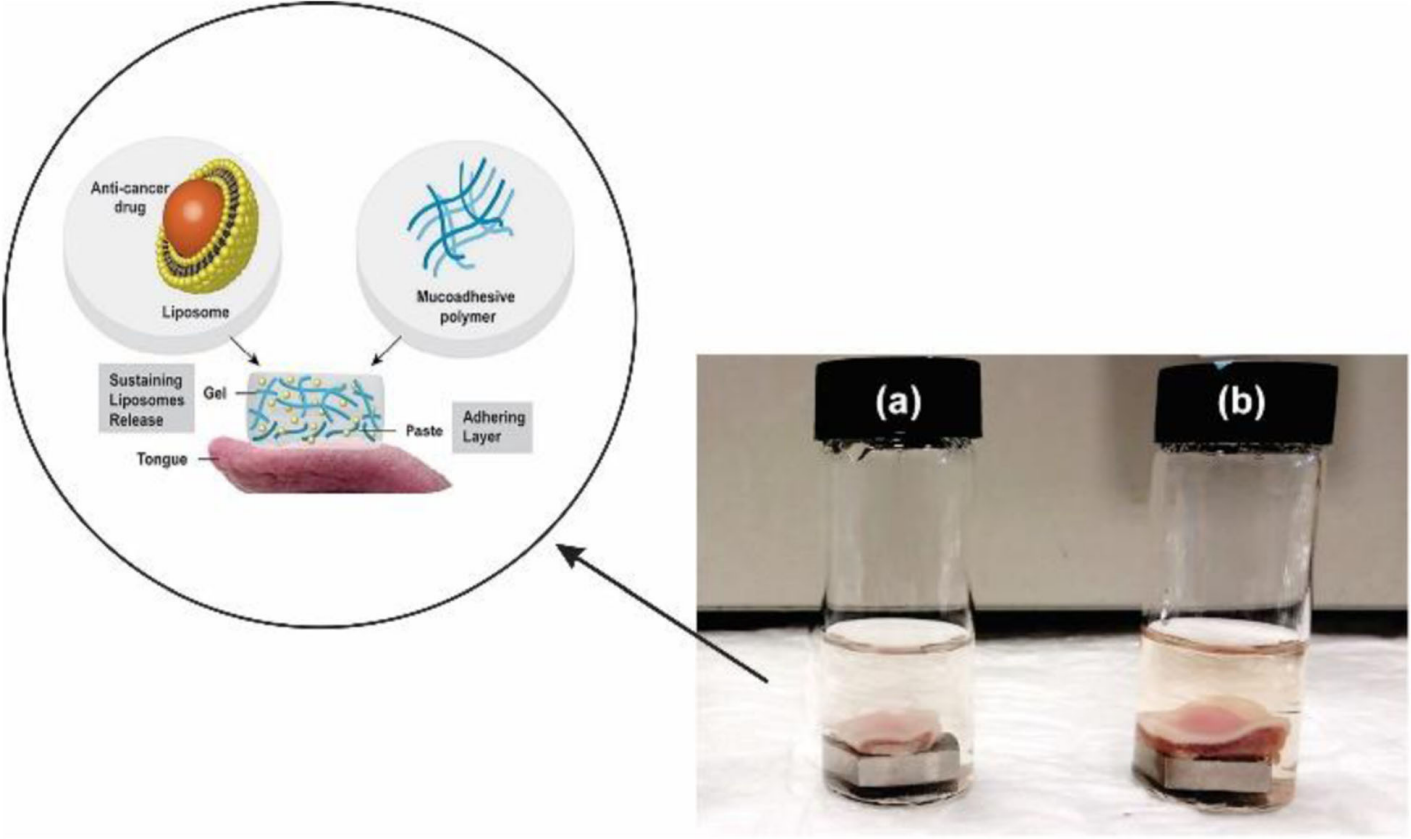
**a** Schematic representation of Alginate-Gelatin composite generated with the Schiff- base reaction. **b** Illustration of the self-healing process in the Oxidized Alginate-Gelatin type B (OxA-GB) hydrogel. The bottom left image depicts the crosslinked hydrogel cutting in three pieces. The bottom right image shows the stretching of the hydrogel after the recoupling of the pieces (optimum healing time: 7 days). **c** Schematic representation of the OxA crosslinking with gelatin in the presence of borax [64]. Pettignano, A., et al., Self-healing alginate–gelatin biohydrogels based on dynamic covalent chemistry: elucidation of key parameters. Materials Chemistry Frontiers. 2017, 1(1):73–79. Copyright (2020)

Fabrication and design of the biodegradable hydrogels, by utilizing the click reactions, developed the extensively science 2001 [59]. Those reactions have advantages such as high yields, fewer by-products, high selectivity, and specificity. The most common example is, copper (I) catalyzed 1,3-dipolar cycloadditions between azides and alkynes. However, the intrinsic toxicity of the transition metals limits the application of the above reaction in the regenerative medicine [60]. There are some metal-free alternative methods for click conjugation including Diels-Alder [61], Schiff base, Oxime and Michael addition [62]. In 2014, a composite of the alginate-gelatin was developed on the basis of a Schiff-base reaction between the oxidized alginate groups and amino gelatin groups. Sodium periodate oxidized the alginate to the alginate dialdehyde (ADA) which could easily crosslink to amino groups of gelatin via Schiff- base reaction (Fig. 9) [63]. Schiff-base which is the so-called pseudo-covalent bond takes the advantages of the dynamic equilibrium between Schiff-base linkages and the aldehyde and amine reactants. Therefore, due to the uncoupling and recoupling of the imine linkage, self-healing property appears in the hydrogel network. Dynamic covalent chemistry was also utilized in the fabrication of the biohydrogels with self-healing properties [64]. Through the present study, gelatin type B was cross-linked to the oxidized alginate (OxA) in the presence of the borax which produced a hybrid biohydrogel system (OxA-GB) (Fig. 10). A major goal in designing the alginate-based hydrogels is being injectable at or below the room temperature, biodegradable, biocompatible and appropriate support for cell induction.

## Alginate applications

### Alginate-based drug delivery vehicles for cancer treatment

Cancer is a major public health problem worldwide. It includes a wide range of diseases arise from the uncontrolled growth of the malignant cells which have the metastatic ability in the body [65]. Considering more than 10 million new cases each year, the World Health Organization predicted cancer-related deaths by about 13 million by the year 2030 [66].

However, thanks to the better understanding of tumor biology, improved treatment methods and diagnostic devices, the mortality rate has decreased through the past 5 years. Chemotherapy, radiation therapy or a combination of them are common methods that currently used in the cancer treatment. Chemotherapy can mainly affect DNA synthesis and mitosis which results in the death of rapidly growing and dividing cancer cells. Current cancer treatment options include the surgical intervention, chemotherapy, and radiation therapy or a combination of these options. The agents of the chemotherapy are nonselective and cause severe systemic toxicity which leads to the unintended side effects in the healthy tissues, e.g., loss of appetite and vomiting, though. On the other hand, because of the poor bioavailability of the mentioned drugs to the tumor site, high doses of them are required, which leads to the enhanced toxicity to the normal cells and increased occurrence of the multiple drug resistance. In fact, these severe side effects induced by the chemotherapeutics on normal tissue and organs are the major causes of the high mortality rate of the cancer patients. Therefore, developing the different delivery systems which can target cancer cells either actively or passively, thereby reducing adverse side effects to the normal tissues, is desirable [67]. Recently, a variety of delivery systems with improved therapeutic efficacy have developed due to the understanding of the tumor biology and developing versatile materials with increased bioavailability such as polymers [68–70], lipids [71, 72], polymeric hydrogels [73, 74], inorganic carriers [75], and biomacromolecular scaffolds [76]. The entrance of the nanotechnology in the field of the clinical therapeutics has had grate impact during the last two decades. In contrast to the conventional chemotherapeutic agents, nanoscale delivery systems have the potential to improve the treatment efficacy while avoiding systemic toxicity via enhanced permeability and retention (EPR) effect and active cellular uptake [77, 78]. Among versatile drug carriers, polymeric hydrogels are the issue that needs to be studied more since their 3D interconnected structure with high capacity of water absorption makes them similar to the human soft tissue microenvironment. Hydrogels can be designed either in the form of the continuous microscopic networks, named macrohydrogels, or discrete particles. In the latter case, if their dimensions are in microscale (above 1 μm), they are called microgels [79]. However, when the particle sizes reach the submicron ranges, they are known as nanogels [79]. Thus, nanogels (NG) are physically or chemically cross-linked hydrophilic three-dimensional polymer networks with sizes up to a few hundred nanometers that swell in water [79–81]. As reported previously, nanogels could also be used as drug/gene carriers since they have good stability in the biological fluid as a result of low deriving force for their aggregation [82]. In comparison to the other nanocarriers, NGs have excellent biocompatibility, high water dispersibility [79]. They also have easy drug loading ability and multiple stimuli-response characteristics (caused by pH, temperature, redox and/or enzyme in targeted sites) [83]. Those outstanding benefits make them suitable systems to load variable therapeutic agents through the physical encapsulation or chemical conjugation [84]. Also, their flexibility could prolong their circulation lifetime via reducing the possibility of their capture by macrophages compared with the corresponding rigid nanoparticles [85]. In generally, their controllable architecture and cell-mediated characteristics make NG an appropriate nanocarrier for in-vivo delivery of drugs/ nucleic acids in treating many diseases such as cancer, neurological disorders, bone degeneration, etc. [86, 87].

Alginate hydrogels have outstanding properties such as high-water content, nontoxicity, soft consistency as well as biocompatibility and biodegradability which make them suitable candidates as drug carriers to deliver the low molecular weight drugs and macromolecules including proteins and genes either sustain or localized [88]. The cargos could immobilize or encapsulate in pores of the carriers. Depending on the pH of the surrounding medium, ALG could form two types of gels. At low pH (gastric environment) it shrinks and produces a viscose acidic gel which does not release its encapsulated drugs. Once it passed through the intestinal tract with higher pH, the skin-like structure of alginic acid converted to the soluble viscose gel, in which the disruption of the polymeric network causes drug dissolution and release. So, in case of delivering the drugs to the target tissue, controlling release over a sustained period of time avoid systemic toxicity. The drug releases from the pores of the hydrogel are carried out by the various mechanisms including diffusion-controlled, swelling controlled, chemically controlled and environmentally-responsive release [89]. In diffusion-controlled release systems, the reservoir or matrix devices are utilized to control the drug release via diffusion from the hydrogel mesh or the pores filled with water. A reservoir delivery system includes a core containing a hydrogel membrane, generally available as capsules, spheres or slabs, cylinders. The drug concentration is very high at the center of the system so that it favors the sustained release of drugs [90]. Alginate hydrogels could be used in pharmacology as emulsions stabilizers, suspenders, tablet binders and disintegrating agents for tablets [91]. Hollow microcapsules based on the alginate composites could be considered as drug delivery vehicles. They usually constructed with the layer-by-layer technique which is a series of negatively and positively charged polyelectrolytes self-assembly. For example, the alternative decomposition of the alginate/chitosan onto CaCO_3_ particles, followed by the removal of the core to obtain the hollow microcapsule with great biocompatibility and ability to load positively charged substances. Table 1 summerizes some sexamoles of alginate-baset drug delivery systems.

**Table 1:** Various alginate-based drug delivery vehicles used in cancer therapy

Description of Carrier	Type	Overcomes multi-drug resistance	Drug	Specify	Ref.
Chitosan-alginate polyelectrolyte multilayer capsule filled with bovine serum albumin gel (BSA-gel-capsule	Microcapsule	Local chemotherapy against drug-resistant (the treatment of drug-resistant breast cancer)	DOX	drug-resistant breast cancer (MCF-7 and MCF-7/ADR)	[92]
lectin-conjugated chitosan–Ca–Alginate	Microparticles	Deliver the drug molecules to colon region, and improve the efficacy in targeted anticancer colon drug therapy	5-FU	Colon cancer (Caco-2)	[93]
Alginate-g-Poly(N-isopropylacrylamide) (alginate-g-PNIPAAm)	Injectable Hydrogel	Sustained release and effective delivery of anti-cancer drugs, overcoming the multidrug resistance in cancer treatment	DOX	prostate cancer (AT3B-1)	[94]
Alginate- Cyclodextrin	Nanogel	Enhance chemotherapeutic efficacy by pressure-controlled drug release	5-FU	Colon cancer (HT-29)	[95]
Magnetic Alginate/Chitosan	Nanoparticles	Sustained release profiles, enhanced uptake efficiency, strong cytotoxicityto cancer cells, potential for targeted drug	Cur	Human breast cancer (MDA-MB-231)	[96]
Folate conjugated hyaluronic acid coated alginate	Nanogels	Antitumor and apoptosis efficacy on colon cancer therapy	OXA	Colorectal cancer (HT29)	[97]
Alginate-keratin composite	Nanogels	Better anti-tumor effect and lower side effects	DOX	Breast cancer (4T1 and B16)	[98]
Alginate nanogel platform	Nanogels	Inhibit tumor growth, reduce the adverse side effects, improve the quality of life of cancer patients.	Cisplatin/ Gold	Breast cancer (MCF-7)	[99]
Hybrid alginate/liposomes hydrogels	Liposome	Enhance chemotherapeutic efficacy by controlled drug release in oral cavity	DOX	Human tongue carcinoma (CAL-27)	[100]
Alginate-PAMAM (G5) hybrid nanogel	Dendrimer	Sustained release, targeted and sufficient tumor an accumulation, increased efficacy and decreased toxicity	EPI	Human breast cancer (MCF-7)	[101]
Dual crosslinked methacrylated alginate (Alg-MA	Sub-microspheres	Increase the efficacy of cell internalization and bioactivity of DOX-loaded Alg-MA, decreased in systemic toxicity of free drug	DOX	Human lung epithelial carcinoma cells (A549s)	[102]

**Fig. 11:**
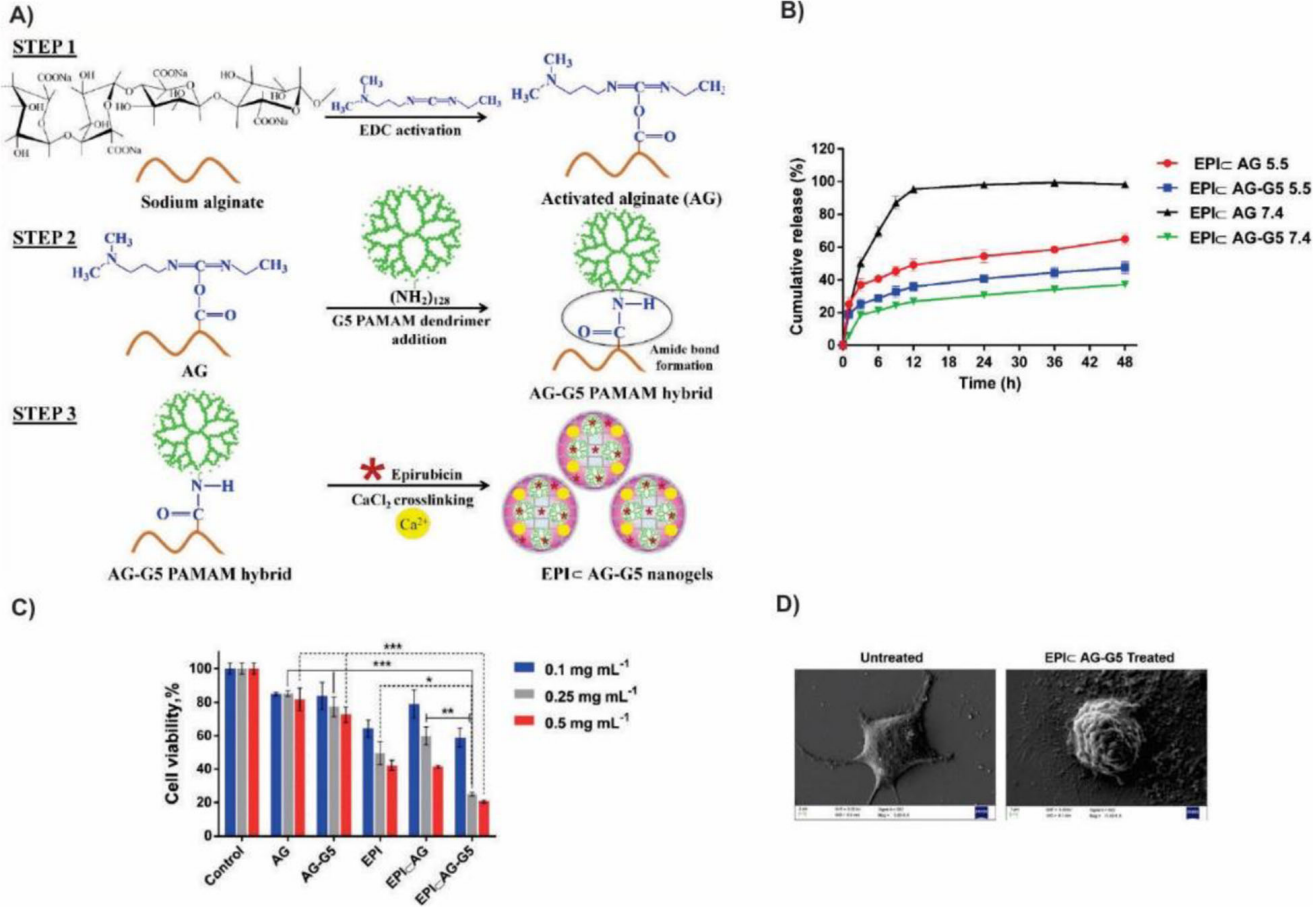
Schematic of DOX loaded hybrid cross-linked alginate/liposome past on tongue tissue and displaying deferent hybrid systems of alginate with Rhodamine labeled liposomes (pink) after 2 h release. **a** past **b** cross-linked past [100]. Shtenberg, Y., et al., Mucoadhesive alginate pastes with embedded liposomes for local oral drug delivery. International journal of biological macromolecules. 2018, 111:62–69. Copyright (2020)

In 2017 Shtenberg and co-workers developed an appropriate hybrid of alginate and liposome as an innovative carrier in oral mucoadhesive drug delivery system. Alginate induces adhesive property and local release of the drug while liposome improves the absorption of the drug into the cells and preserve from degradation. To investigate the release kinetics of liposome loaded anti-cancer drug doxorubicin (DOX), three alginate/liposome combination was investigated. A hybrid past with perfect adhesive properties but fast burst release (90% after 2 h); a hybrid hydrogel with controllable release rate (5, 30% or 60% after 2 h) but poor mucoadhesive capability; and finally, a hybrid cross-linked past with controllable release rate of 20% after 2 h were developed (Fig. 11). In adherence studies, the retention of polymer on tongue tissue during a long time was 50% for the past and 80% for the cross-linked past verifying stronger adhesion of cross-linked past [100]. Anti-tuberculosis drugs were tested in mice treated with cation-induced calcium chloride alginate nanoparticles. The concentrations of therapeutic drugs were taken for 7–11 days at an oral dose in the blood plasma, and a total of 15 days in organs such as the lungs, liver, and spleen. The drugs encapsulated in nanoparticles are highly bioavailable toward to the free drugs. Also, in the mice infected with M. Tuberculosis, for complete clearance of bacterially infected organs needed just three oral doses of the nanoencapsoulated drug that takes15 days, while 45 doses of the free drug daily which confirmed the slow and sustained release of nanoencapsoulated drug [103].

Among cancer-related death, breast cancer is the most common leading cause of death among women. It is estimated by the experts more than 268,600 new cases with invasive carcinoma and 62,930 new cases with non-invasive carcinoma will be affected in the U.S. in 2020 [104]. Also, about 41,760 women are expected to die from the breast cancer in the U.S. in 2020. Earlier diagnosis and better adjuvant therapy played an important role in the improved patient outcomes. A group of the researchers in 2015 promoted the alginate-PAMAM dendrimer (AG-G5) hybrid nanogel as a suitable platform for the enhanced anti-cancer drug delivery (Fig. 9). To embedding of PAMAM onto the alginate backbone, they utilized 1-ethyl-3-(3-dimethylamino propyl) carbodiimide hydrochloride (EDC) for the alginate carboxylate groups activation to form the amide bond with PAMAM amine groups. Finally, the remained carboxylate groups of the alginate networks were cross-linked in the presence of the calcium chloride (CaCl_2_). Therefore, the combination of the ionic and covalent bonds in the alginate-PAMAM dendrimer network caused to enhanced the structural stability and drug encapsulation efficacy. They used EPI as a model drug for encapsulation in AG-G5 nanogel and examined the anti-cancer efficacy of the resulted EPI & AG-G5 nanogel in MCF-7 human breast cancer cells. The penetration of the G5 PAMAM within the AG network reduced the size of the nanogel and impart superior responsiveness to the nanocarrier. Also, EPI & AG-G5 was capable to release its payload in a sustained and controlled manner. Furthermore, the in-vitro cytotoxicity study of the EPI & AG-G5 nanogels indicated that it could induce the cell death through apoptosis [101].

**Fig. 12:**
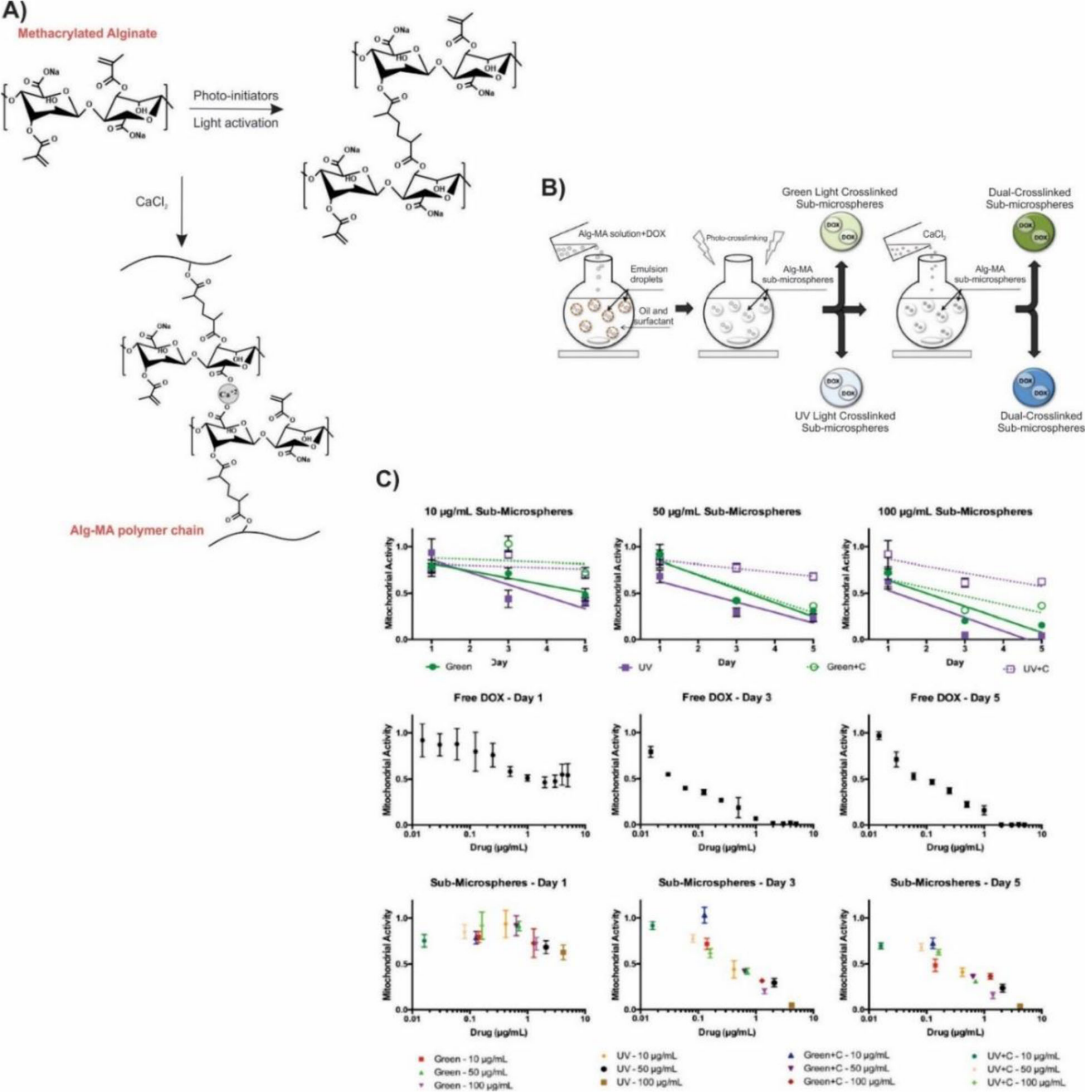
Cytotoxicity assessment of DOX using dual-crosslinked Alg-MA sub-microspheres as chemotherapeutic delivery vehicles. **a** Chemical structure of dual-crosslinked Alg-MA hydrogel networks. **b** Schematic representation of microsphere fabrication techniques. Premixing of Alg-MA solutions with or without DOX was followed by water/oil emulsion at room temperature generated microspheres. Alg-MA sub-microspheres were photo-crosslinked upon the exposure to visible or UV light, respectively, and further dual-crosslinked in the presence of 1 M CaCl_2_. **c** MTT-based assay of DOX loaded dual-crosslinked Alg-MA sub-microspheres to quantify the cell proliferation over a 5-day period. A549 activity was recorded as the mitochondrial activity and normalized to the non-modified cell controls. Various formulations and concentrations (10–100 μg/mL) of the sub-microspheres were assessed: green photo-crosslinked (Green), green + Ca^2+^ dual-crosslinked (Green+C), UV photo-crosslinked (UV), UV + Ca^2+^ dual-crosslinked (UV + C). DOX was added exogenously (Free DOX) to the cell culture medium at the various concentrations to test the effects of the intracellular versus extracellular DOX delivery [107]. Fenn, S.L., et al., Dual-cross-linked methacrylated alginate sub-microspheres for intracellular chemotherapeutic delivery. ACS applied materials & interfaces. 2016, 8(28):17775–17,783, Copyright (2020)

Lung cancer is the most typical cancer among men and women and one of the cancer-related causes of the death in the world [102, 105]. Over 85% of lung cancer cases categorized as non-small-cell lung cancer (NSCLC), including adenocarcinoma, squamous-cell carcinoma, and large-cell carcinoma [102]. Despite the recent development of the lung cancer detection and treatment, NSCLC is usually diagnosed at an advanced stage and has a poor prognosis. As a result, the five-year-survival rate of lung cancer is about 18% [106]. In another study, to overcome the limitations associated with alginate sub-microspheres as drug carriers including low drug encapsulation efficacy and rapid drug release rate (< 24 h), Fenn et al., generated the dual-crosslinked methacrylate alginate (Alg-MA) sub-microspheres by using water/oil emulsion and subsequent crosslinking (Fig. 12). The irradiation of visible (green) or UV light leads to covalently crosslinking Alg-MA sub-microspheres formation. The subsequent addition of the CaCl_2_ generated the dual-crosslinked sub-microspheres. To evaluate the Alg-MA sub-microspheres as chemotherapeutic delivery vehicles, they utilized DOX as a model drug for its intrinsic UV absorption. Also, they assessed the efficacy of the cell internalization and bioactivity of DOX-loaded Alg-MA sub-microspheres on the human’s lung epithelial carcinoma cells (A549s). The results of the MTT suggested the successful encapsulation of the DOX by photo crosslinked and dual-crosslinked Alg-MA as well as internalization to the A549 cells which reduced the mitochondrial activity in comparison with the untreated cells. Finally, they achieved to the effective and controllable clinical drug dosages as compared to the free DOX delivery based on the drug encapsulation predictions and calculations [107].

### Other applications

#### Wound dressing

Wound dressings were used for the treatment of the severe skin burns and injuries for centuries. The primary benefits of the traditional gauze-based dressings were easy to handle, great ability to absorb and reasonable price. However, peeling off gauze secondary injury could occur. Currently, the high-quality dressings for a wound are considered as an active component of the healing process that is designed to control the infection, stop bleeding, absorb exudates, permeable to water and gas transformation and provide a warm, moist environment for the fast and effective healing process. Among several dressing materials, hydrogels are promising candidates used for the treatment of burns and chronic wounds [108]. Alginate, a naturally derived hydrogel, is extensively used for dressing the production because of the properties such as biodegradability, nontoxicity, excellent water absorption capacity, easy to use, hemostatic property and non-immunogenicity [109–111]. Alginate dressings are constructed by the gel formation through ionic cross-linking of its solution with Ca, Mg, Ba, Zn, etc. as well as freeze-drying of porous sheets in the form of foam or fibrous dressing [30, 112]. They could absorb the wound excaudate in both gel and dry form while providing a physiologically moist environment and minimized bacterial infection. The amount of M-block in the alginate structure affected the immunogenic property by favoring the cytokines production [22]. The process of healing is carried out by enhancing the monocytes to generate the high levels of cytokines such as interleukin-6 and factor-α tumor necrosis which in turn stimulate the anti-inflammatory factors [113].

Biocompatibility, porosity, high water content, and permeability to water and gas are some of the significant properties that make alginate hydrogel an ideal candidate for wound dressing. In spite of these outstanding properties, it still suffers from shortcoming such as poor mechanical stability in swollen form and easily dehydration if not covered with a secondary dressing [114]. The composition of alginate with some synthetic or natural polymers could improve its mechanical stability.

In the treatment of the chronic injuries with exudate or infected surgical wounds, a dressing contains calcium alginate is capable of the ions transferring with the wound fluid in order to help the blood clotting and act as a hemostat [115]. The resulted soluble and absorbent gel provides a moist environment and helps the healing process by assisting the fresh epidermis to develop [75, 79]. A group of researchers in 2012 produced the hydrogels of sodium alginate (SA) and gelatin (G) type B in ratios that are shown in table x-x for potential application as a wound dressing. The morphology of the hydrogels was influenced by the sodium alginate and gelatin ratios. The micrographs of SEM showed that when the composition of SA/G was 70/30, 60/40 and 50/50 the morphology of hydrogels was in droplet form while hydrogels with a ratio of 40/60, 30/70 and 20/80 represent fibrous morphology. The composition of the prepared hydrogels affected the swelling behavior which came from functional hydrophilic groups of a polymer such as -COO^−^ and -NH_2_ [116].

**Fig. 13:**
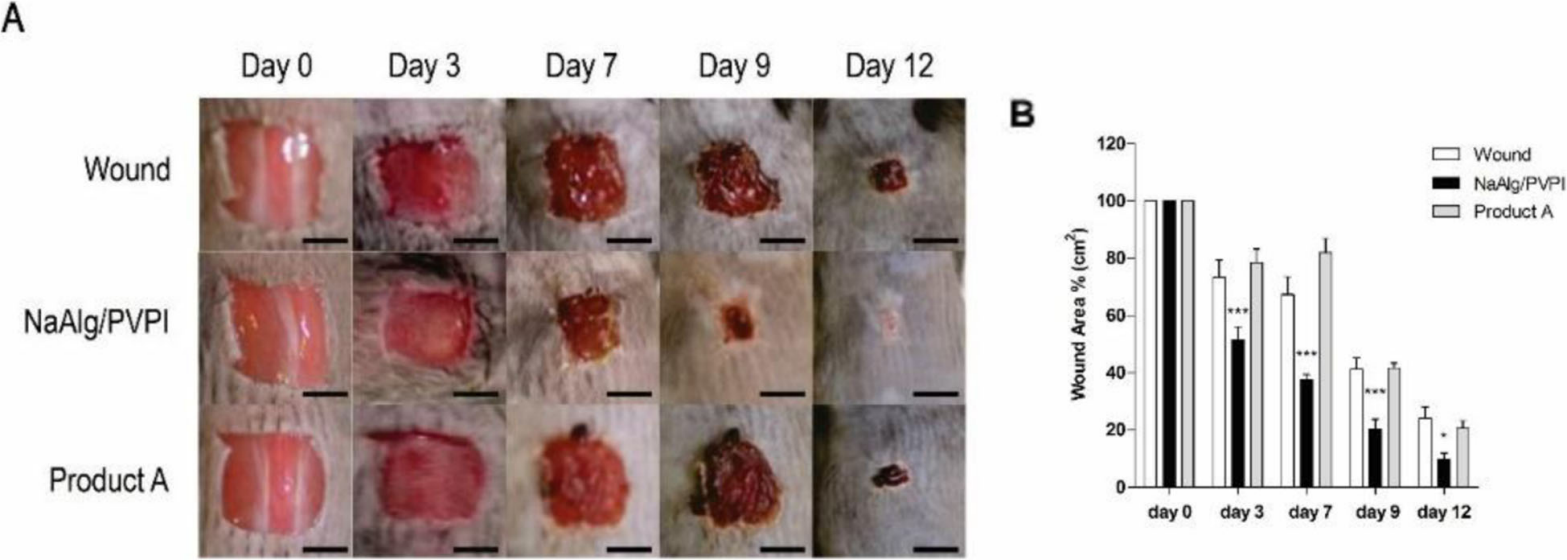
**a** Representative photographs of wound healing mice skin at different times (day 0, day 3, day 7, day 9, and day 12) for untreated wound (no dressing), NaAlg/PVPI and Product A (A commercial Povidone Iodine Non-Adherent Dressing product) as control sample are shown (scale bar 5 nm) **b** Days of wound healing in mice untreated (white bars), and treated with the NaAlg/PVPI (black bars) or with Product A dressing (grey bars). *p < 0.05 and ***p < 0.001, compared to the untreated mice [117]. Summa, M., et al., A biocompatible sodium alginate/povidone iodine film enhances wound healing. European Journal of Pharmaceutics and Biopharmaceutics. 2018, 122:17–24. Copyright (2020)

In a recent investigation, a composition of the sodium alginate (NaAlg) and an antiseptic agent povidone-iodine (PVPI) was prepared to aim to provide a wound dressing with great healing performance due to the alginate as well as bactericidal and fungicidal properties of povidone-iodine (Fig. 13). The encapsulation of PVPI in the alginate polymeric network was resulted from the controlled release of the mentioned agent and avoided its possible toxicity. The in-vitro and in-vivo studies were led to characterize the efficacy of the composite in the healing process. Furthermore, the NaAlg/PVPI composite displayed the excellent biocompatibility, biodegradability, reducing the inflammatory response, rising proline levels as a collagen content indicator, resulting in the reduced re-epithelialization time in the skin wound model of the mouse [117].

In general, the swelling capacity of the alginate-based hydrogels is affected by polymer composition [116, 117] and the presence of nanoparticles or plasticizers [118, 119]. Increasing the swelling ability will provide a moist environment for the wound, as a result, reduce bacterial infection and improve the healing process. In addition, incorporating antibacterial agents [120], nanoparticles [121] and coating of hydrogels with honey [122] or chitosan [123] induce antibacterial property to the alginate hydrogels.

#### Alginate based bioink in 3D bioprinting

Since the 1950s, millions of patients with incurable diseases were survived through the organ transplantation. The available donors; however, were less fewer than the growing demand for this procedure. On the other hand, the organ transplantation had limitations on the immune response and organ rejection. As an alternative to the organ transplantation, 3D bioprinting technology has been developed significantly since the first U.S. patent award in order to provide the practical and promising outcomes in the field of regenerative medicine. In this regard, a lot of attempts have been made, from bioprinting of cells and biological molecules to biomanufacturing of the tissues and organs.

3D bioprinting tissue engineering technology provides layer by layer printing of bioink in a scaffold-free manner to mimic the structure of living tissue. The mentinoned technique makes it possible to generate 3D, scalable and complex geometry with spatial heterogeneity that is not afforded by the scaffold-based technique.

**Fig. 14:**
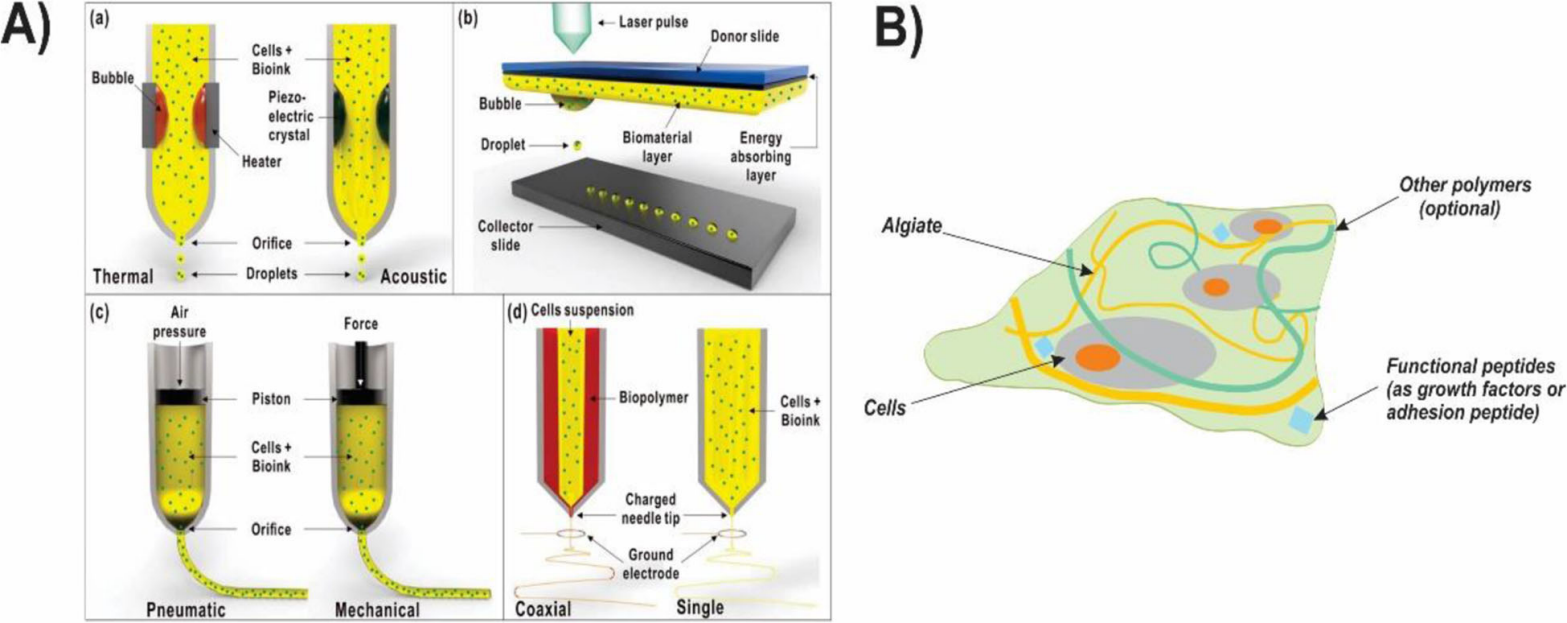
A) Different types of bioprinting techniques and their application in organ systems. (a) Inkjet bioprinting method (b) Laser-assisted bioprinting method (c) Extrusion bioprinting method (d) Bio-electrospraying/Cell electrospinning [124]. B) The illustration of the bioink based on the alginate (composed of the cells, alginate hydrogel, and—optionally—functional peptides to improve the cell’s biological function) [126]. Republished with permission of ref. [124], Hong, N., et al., 3D bioprinting and its in vivo applications. Journal of Biomedical Materials Research Part B: Applied Biomaterials. 2018, 106(1): 444–459, Copyright (2020)

A 3D bioprinted tissue or organ produced either in-vitro that incubated in the bioreactor for maturation before implantation by surgery or in-situ in which the human body act as a bioreactor. A variety of studies have been made for bioprinting tissues such as bone, cartilage, skin, vascular and human-scale ear cartilage. Furthermore, studies have extended to other research areas such as drug delivery. Some of the typical strategies in 3D bioprinting include the extrusion, inkjet, layer assisted and cell electrospinning (Fig. 14).

Among the several biopolymers, hydrogels are promising candidates as a matrix in bioink. Bioink is commonly referred to the biomaterials which could encapsulate the cells and are printable into three-dimensional scaffolds and tissue-like structures. They are enabled to mimic or replace the target tissue upon their similarity and their tunable degradation to the native extracellular matrix (ECM) and physical properties. Their viscosity and the procedure which transforme the sol to gel determined the structure and shape fidelity of the resulted three-dimensional bioprinted hydrogels. Hydrogels with a jelly-like structure mostly are consist of water and don’t exhibit any stream in their steady-state thanks to the cross-linked polymer network inside the fluid, and as a result, they find properties similar to the human tissues [124, 125]. Several biocompatible hydrogels are used as bioinks to develop the cell proliferation in 3D bioprinting techniques such as gelatin, agarose, hyaluronic acid, and alginate [126].

**Fig. 15:**
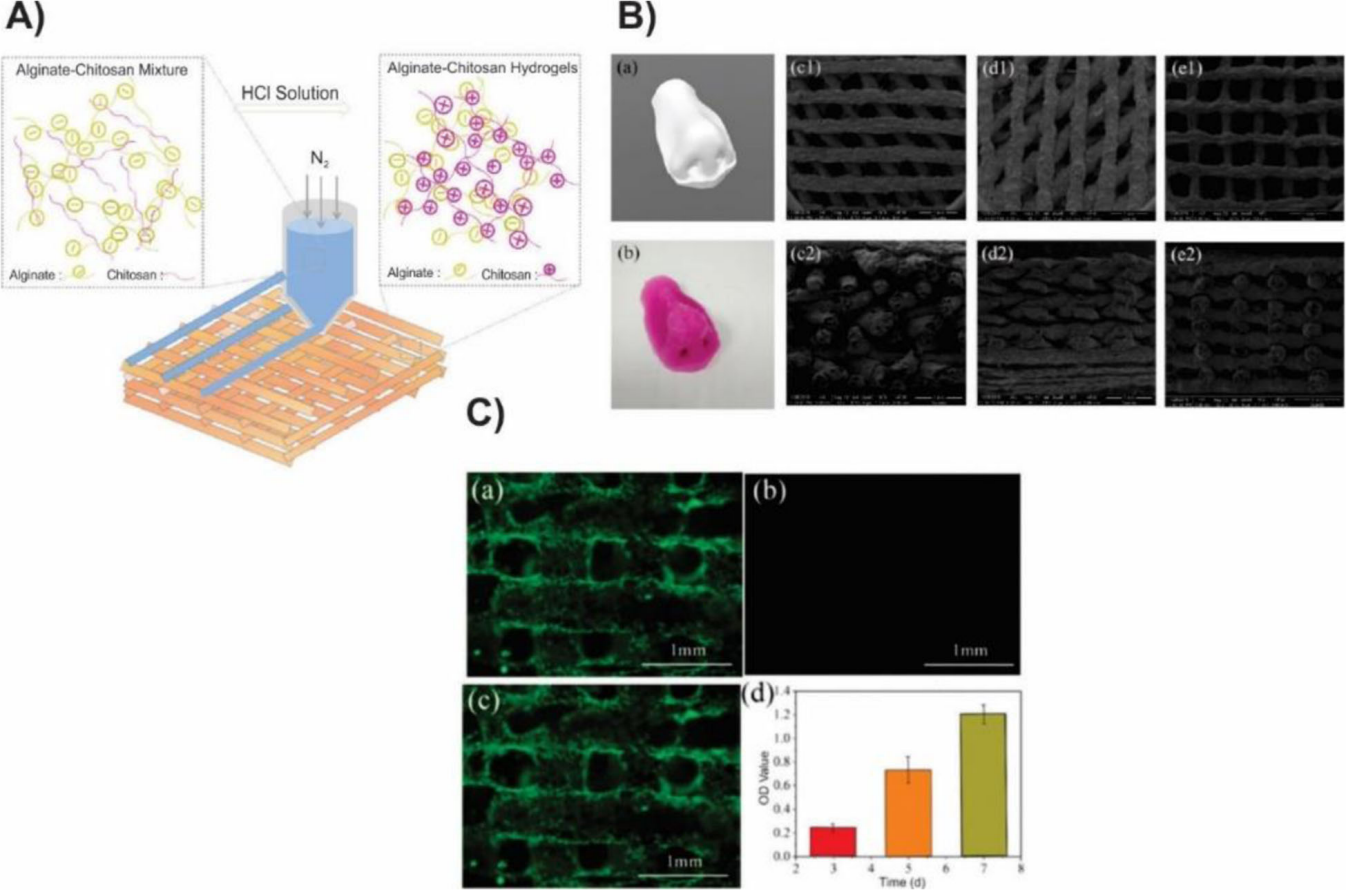
A) Schematic illustration of the alginate-chitosan (Al Ch) polyionic complex hydrogel as bioink in 3D bioprinting. B) Morphological characterization of 3D bioprinted Al Ch a) 3D model of nose b) 3D printed nose, constructed by Al1Ch1.2 bioink and SEM micrograph of 3D printed Al1Ch1.0 bioink with different angles between the filaments (C1, C2) 45°, (d1, d2) 60°, (e1, e2) 90° (1,2 respectively represent front and side of the scaffold) biocompatibility of the 3D bioprinted AlCh polyionic hydrogel. C) Human adipose-derived stem cells (hASCs) were used to test the biocompatibility of the scaffold. Photographs of the inverted fluorescence microscope which represent a) live b) dead c) merged cells on the 3rd day. As shown in the pictures, the live cells distributed uniformly on the hydrogel while little or no dead cells existed. d) proliferation of hASCs distributed on the 3D bioprinted hydrogel. It showed that hASCs could proliferate during the time [127]. Liu, Q., et al., Preparation and Properties of 3D Printed Alginate–Chitosan Polyion Complex Hydrogels for Tissue Engineering. Polymers. 2018, 10(6):664.Copyright (2020)

Sodium alginate, a naturally occurring polyanionic and linear block copolymer exhibits the high biocompatibility due to the supporting of cell growth. Thanks to the shear-thinning property, alginate solution is an ideal precursor for 3D bioprinted tissue-engineered constructs (Fig. 14) [30]. To overcome the in-situ cross-linking limitations, alginate solution engineered to be shear-thinning in which it could decrease its viscosity with increasing shear. Therefore, alginate hydrogels could be extruded from the syringe upon utilizing shear and immediately reform when the mechanical force stopped. In case, that alginate solutions are used as the bioink to prepare the scaffolds, the structure and shape fidelity of the resulted hydrogels are tough to be guaranteed as a result of the insufficient viscosity comes from the maximum concentration of the alginate solutions which leads to the ease of the collapse and fused of the deposited filaments. Particularly when the alginate hydrogels cross-linked with Ca^+ 2^, they become mechanically poor and easily collapsed with the gravity which affects their structure and shapes the fidelity of three-dimensional printed hydrogels. One of the challenging issues in using the alginate hydrogels as bioink in 3D bioprinting is its low rate degradation, which could be tailored by oxidation (e.g. through Na_2_O_2_) or by altering the γ-ray molecular weight distribution of the alginate. The degradation process was also catalyzed by alginate lyase. By dispersing the chitosan powder in the alginate solution, Liu et.al improved the viscosity of the solution 1.5–4 times (Fig. 15). In that way, the shape fidelity of the 3D printed polyionic alginate-chitosan (Al Ch PIC) hydrogels could improve. They produced 3D printed hydrogel by spraying HCl (1 M) between the two deposited layers. The addition of the chitosan to the alginate medium improved 3D bioprinting ink viscosity as its amine groups change into the ammonium groups in the acidic medium and the electrostatic interaction between the two positively and negatively charged polyelectrolytes construct 3D bioprinted alginate–chitosan polyion complex hydrogels. As a result, the deposited filament prepared in that way were not easily collapsed or fused. They used the alginate-chitosan for printing the nose to confirm that the prepared 3D bioprinting ink could be successfully utilized in 3D bioprinting of tissues or organs with complex structures [127].

**Fig. 16:**
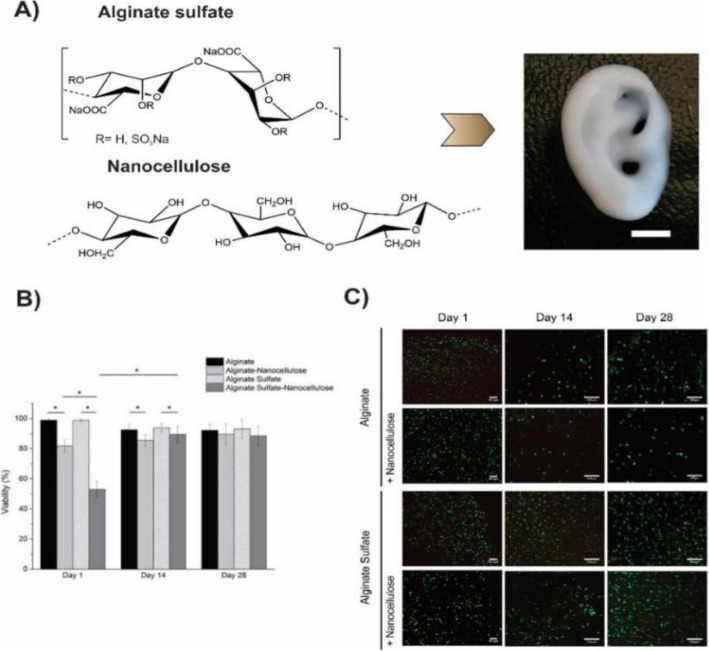
**a** Chemical structure of the alginate sulfate and nanocellulose. Incorporation of the two biomaterials generates an ideal bioink which is appropriate for the 3D bioprinting of the complex constructs. Here a miniature size eare was 3D bioprinted (scale bar is 5 mm). **b** and **c** Viability assessment of chondrocytes and Live/dead staining of bovine chondrocytes respectively, encapsulated in alginate and alginate sulfate with or without nanocelloluse after 1, 14 and 28 days of culture. The scale bar is 100 μm [128]

**Fig. 17:**
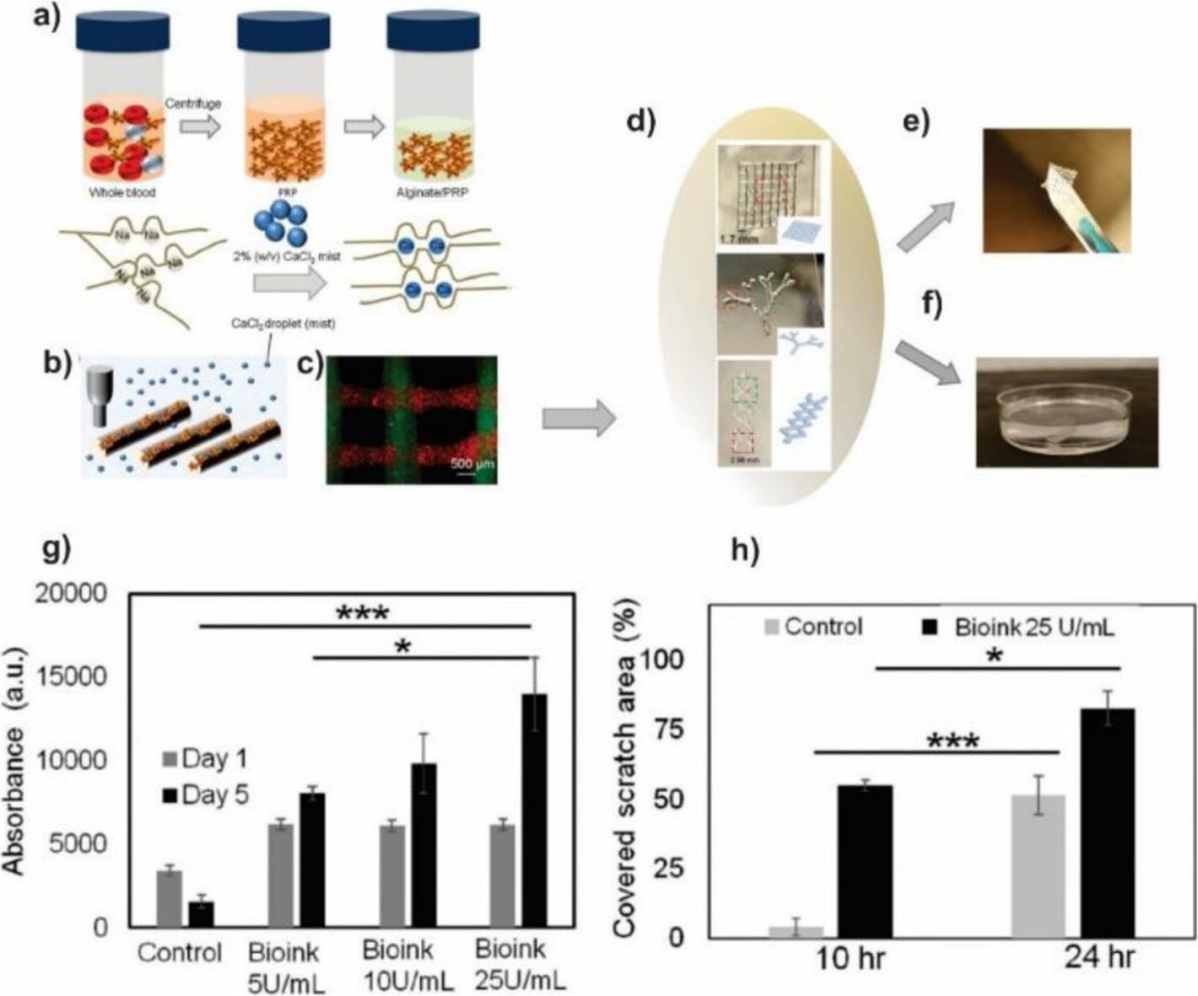
**a** Chemical structure of the alginate sulfate and nanocellulose. Incorporation of the two biomaterials generates an ideal bioink which is appropriate for the 3D bioprinting of complex constructs. Here a miniature size eare was 3D bioprinted (scale bar is 5 mm). **b** and **c** Viability assessment of chondrocytes and Live/dead staining of bovine chondrocytes respectively, encapsulated in alginate and alginate sulfate with or without nanocelloluse after 1, 14 and 28 days of culture. The scale bar is 100 μm

Müller et.al. reported an alginate sulfate-based mitogenic hydrogel and nanocellulose in which alginate sulfate supported the chondrocyte phenotype, and nanocellulose improved the rheological properties of the hydrogel and increased printability of the bioink (Fig. 16). The addition of the nanocellulose to the alginate sulfate solution increased its viscosity 3–7 times depending on the shear rate. Nanocellulose is an appropriate substance to enhance the printability of the low viscosity material owing to its biocompatibility and intrinsic mechanical property. They evaluated the viability of the chondrocytes that were encapsulated in alginate, alginate sulfate, alginate nanocellulose, and alginate sulfate nanocellulose. As depicted in Fig. 17, alginate and alginate sulfate gels showed the good viability, but after the addition of the nanocellulose, the viability decreased in day 1, that probably related to the unknown interaction between alginate sulfate and nanocellulose. However, the innate property of alginate sulfate led to the cell proliferation, and cell viability in the alginate sulfate-nanocellulose improved to the same levels as other conditions at day 28. They concluded that chondrocytes in alginate sulfate-nanocellulose matrix were long-lasting, mitogenic and enable to synthesis collagen II. The optimum conditions of the printing which best preserved cell functions were wide diameter and conical needle [128].

**Fig. 18:**
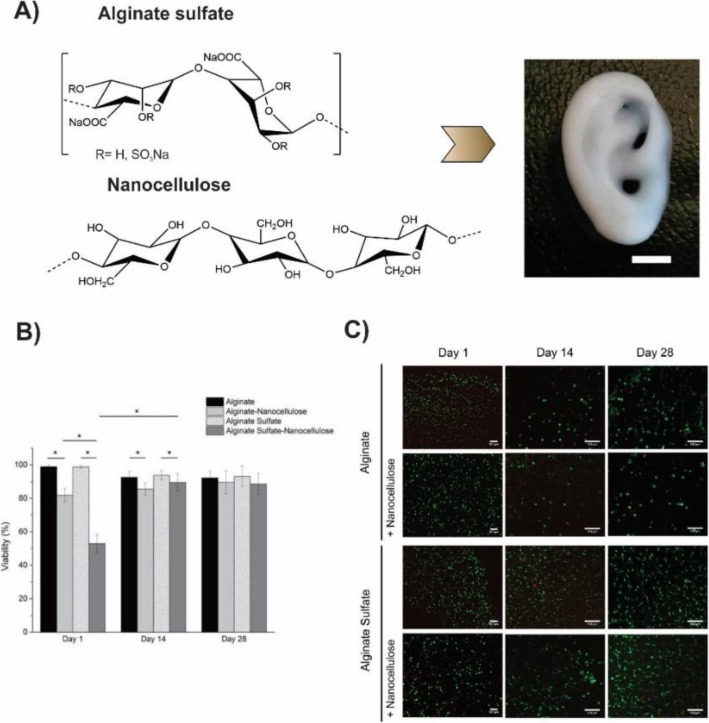
Patient-Specific Platelet-Rich Plasma (PRP) bioink using 3D bioprinting of alginate scaffold **a** Schematic of PRP extraction and its incorporation with alginate to form patient-specific bioink **b** Schematic of proposed bioprinting process **c** PRP incorporated alginate scaffold containing fluorescence particles. **d** Images of different PRP-alginate constructs. In the production of these constructs 0.04% (w/v) CaCl_2_, 50 U ml^− 1^ PRP, and 1% (w/v) alginate was used. **e**, **f** The fabricated constructs could easily be removed from the substrate without losing their integrity. **g** Metabolic activity of mesenchymal stem cells (MSCs) treated with alginate and alginate/PRP over 5 days without any growth factor. **h** Metabolic activity of human umbilical vein endothelial cells (HUVECs) treated with alginate and alginate/PRP over 3 days without any growth factor. (*P < 0.05; **P < 0.01, ***P < 0.001) [129]. Faramarzi, N., et al., Patient-Specific Bioinks for 3D Bioprinting of Tissue Engineering Scaffolds. Advanced healthcare materials, 2018. 7(11), Copyright (2020)

With increasing importance of the personalized medicine, the need to develop bioinks which include specific biological factors of the autologous/patient for tissue engineering and regenerative medicine application is significantly increased. In this regard, Faramarzi et al. used Platelet-Rich Plasma (PRP) as a source of the autologous growth factor and incorporated it with the alginate-based hydrogel (Fig. 18) [129]. PRP contains a high dosage of the platelet and is able to releases a cocktail of growth factors and cytokines in response to injury which leads to induce the healing process. They extracted the PRP from the blood sources of patients to decrease the immune response of the host then mixed with the sodium alginate solution. In order to be printable, the bioink solutions were coated with the calcium chloride agarose gel for 1 h. The resulted hydrogel disk (circular disk diameter 6 mm and height 2 mm) was rinsed with the PBS gently. Rheological characterization showed that the combination of the PRP improved slightly the bioink compressive modulus. Moreover, the presence of the PRP increased the degradation rate and lyophilized cross-linked bioink water absorption capacity. Therefore, the structures that contained PRP had a degradation rate faster than pristine alginate hydrogel and swelled slightly more than pristine hydrogel. The in-vitro studies demonstrated that PRP incorporated bioink could affect positively the mechanism of mesenchymal stem cells (MSCs) and endothelial cells (ECs) involved in the process of tissue healing. They concluded that the prepared bioink might be easily used by any 3D printer based on the extrusion and facilitate autologous and personalized therapies.

## Conclusions

In summary, this review explains the alginate properties, chemical structure, and methods of the hydrogel formation. Alginate has proved great utility and potential as a biomaterial for many biomedical applications including, drug delivery vehicles, wound healing material in wound dressing, and bioink in 3D bioprinting. The most attractive features of the alginate for these applications include biocompatibility, mild gelation conditions, and simple modifications to prepare the alginate derivatives with new properties. Thanks to the properties such as swelling capacity, mucoadhesiveness, and ability of the sol/gel transition, the alginate has gained a preferential place in the development of the drug delivery systems. A chemically modified alginate has been widely used as a carrier to promote the efficacy of the chemotherapeutic agents in cancer treatments. Additionally, the alginate-based hydrogels have applied as a wound dressing due to the improved absorption capacity, mechanical stability, and viscoelastic properties. Due to the excellent biocompatibility, the alginate hydrogels have also demonstrated good printability. It is widely employed through the vascular, cartilage, and bone tissue printing. However, considering the limited mechanical stiffness of the alginate hydrogels, and taking into account variable crosslinking strategies, it is possible to yield hydrogels for each application by using the molecules with the appropriate molecular weights, chemical structures, and crosslinking functionality. The alginate gels have been already used clinically in the wound dressings, but they played passive roles. In future wound dressings, they will likely play more active roles by incorporating the bioactive agents which facilitated the wound healing process with alginate dressing. Although the vast variety of the alginate-based hydrogels have not been successfully used in the clinical application, several new and promising alginate gels with different applications which are currently under development represent a great promise and thus providing hope for new treatment options in near future.

### Statistical analysis

Not applicable.

## Data Availability

Not applicable.

## Abbreviations

5-FU: Fluorouracil
AAD: Adipic Acid Diacid
ADA: Alginate dialdehyde
AlCh PIC: Alginate chitosan polyion complexes
ALG: Alginate
Cur: Curcumin
DOX: Doxorubicin
ECs: Endothelial cells
EDC: 1-ethyl-3-(3-dimethylamino propyl) carbodiimide hydrochloride
EPI: Epirubicin
EPR: Enhanced permeability and retention
GB: Gelatin type B
HUVECs: Human umbilical vein endothelial cells
LCST: Low critical solution temperature
MA: Methacrylate alginate
MSCs: Mesenchymal stem cells
MW: Molecular weight
NaAlg: Sodium alginate
NG: Nanogels
NSCLC: Non-small-cell lung cancer
OXA: Oxaliplatin
OxA: Oxidized alginate
PAMAM: Poly (Amidoamine)
PEG: Poly Ethylene Glycol
PNIPAM: Poly (N-Isopropyl Acryl Amide)
PRP: Platelet-rich plasma
PVPI: Povidone-iodine
RGD: Arginine-Glycine-Aspartic acid
SA: Sodium alginate

## References

[1] Zhu J, Marchant RE (2011). Design properties of hydrogel tissue-engineering scaffolds. Expert Rev Med Devices.

[2] Kumar Giri T (2012). Alginate based hydrogel as a potential biopolymeric carrier for drug delivery and cell delivery systems: present status and applications. Curr Drug Deliv.

[3] Kulkarni RV, Sa B (2008). Enteric delivery of ketoprofen through functionally modified poly(acrylamide-grafted-xanthan)-based pH-sensitive hydrogel beads: preparation, in vitro and in vivo evaluation. J Drug Target.

[4] Kulkarni RV (2010). Interpenetrating network hydrogel membranes of sodium alginate and poly(vinyl alcohol) for controlled release of prazosin hydrochloride through skin. Int J Biol Macromol.

[5] Blandino A, Macias M, Cantero D (2000). Glucose oxidase release from calcium alginate gel capsules. Enzym Microb Technol.

[6] Lamas MC (2001). Calcium alginate microspheres of Bacillus subtilis. Drug Dev Ind Pharm.

[7] Sun J, Tan H (2013). Alginate-based biomaterials for regenerative medicine applications. Materials.

[8] Urtuvia V (2017). Bacterial alginate production: an overview of its biosynthesis and potential industrial production. World J Microbiol Biotechnol.

[9] Smidsrod O, Skjak-Braek G (1990). Alginate as immobilization matrix for cells. Trends Biotechnol.

[10] Rinaudo M (2008). Main properties and current applications of some polysaccharides as biomaterials. Polym Int.

[11] Sachan NK (2009). Sodium alginate: the wonder polymer for controlled drug delivery. J Pharm Res.

[12] Ertesvåg H (2015). Alginate-modifying enzymes: biological roles and biotechnological uses. Front Microbiol.

[13] Maleki S (2017). New insights into Pseudomonas fluorescens alginate biosynthesis relevant for the establishment of an efficient production process for microbial alginates. New Biotechnol.

[14] Firooz A, Bouzari N, Mojtahed F, Pazoki-Toroudi H, Nassiri-Kashani M, Davoudi M, Dowlati Y (2005). Topical immunotherapy with diphencyprone in the treatment of extensive and/or long-lasting alopecia areata 11. J Eur Acad Dermatol Venereol.

[15] Brault D (2003). Methods for obtaining oligomannuronates and guluronates, products obtained and use thereof.

[16] Rahgozar M, Pazokitoroudi H, Bakhtiarian A, Djahanguiri B (2001). Diazoxide, a KATPopener, accelerates restitution of ethanol or indomethacin‐induced gastric ulceration in rats independent of polyamines. Journal of Gastroenterology and Hepatology.

[17] Estrela N (2015). Sucrose prevents protein fibrillation through compaction of the tertiary structure but hardly affects the secondary structure. Proteins.

[18] Fu S (2011). Relevance of rheological properties of sodium alginate in solution to calcium alginate gel properties. AAPS PharmSciTech.

[19] Toroudi HP, Javedan G, Shidfar F, Davoodi SH, Ajami M, Gorjipour F, Sureda A, Nabavi SM, Daglia M (2016). Conjugated linoleic acid rat pretreatment reduces renal damage in ischemia/reperfusion injury: unraveling antiapoptotic mechanisms and regulation of phosphorylated mammalian target of rapamycin. Mol Nutr Food Res.

[20] Kuo CK, Ma PX (2001). Ionically crosslinked alginate hydrogels as scaffolds for tissue engineering: part 1. Structure, gelation rate and mechanical properties. Biomaterials.

[21] Crow B, Nelson K (2006). Release of bovine serum albumin from a hydrogel-cored biodegradable polymer fiber. Biopolymers.

[22] Szekalska Marta, Puciłowska Agata, Szymańska Emilia, Ciosek Patrycja, Winnicka Katarzyna (2016). Alginate: Current Use and Future Perspectives in Pharmaceutical and Biomedical Applications. International Journal of Polymer Science.

[23] Augst AD, Kong HJ, Mooney DJ (2006). Alginate hydrogels as biomaterials. Macromol Biosci.

[24] Singh B, Sharma V, Chauhan D (2010). Gastroretentive floating sterculia–alginate beads for use in antiulcer drug delivery. Chem Eng Res Des.

[25] Jørgensen TE (2007). Influence of oligoguluronates on alginate gelation, kinetics, and polymer organization. Biomacromolecules.

[26] Mohammadhosseini Majid, Sarker Satyajit D., Akbarzadeh Abolfazl (2017). Chemical composition of the essential oils and extracts of Achillea species and their biological activities: A review. Journal of Ethnopharmacology.

[27] Hay ID (2010). Bacterial biosynthesis of alginates. J Chem Technol Biotechnol.

[28] Rubinstein M, Colby RH (2003). Polymer physics.

[29] Hennink WE, van Nostrum CF (2012). Novel crosslinking methods to design hydrogels. Adv Drug Deliv Rev.

[30] Lee KY, Mooney DJ (2012). Alginate: properties and biomedical applications. Prog Polym Sci.

[31] Tan H, Marra KG (2010). Injectable, biodegradable hydrogels for tissue engineering applications. Materials.

[32] Gan T, Zhang Y, Guan Y (2009). In situ gelation of P (NIPAM-HEMA) microgel dispersion and its applications as injectable 3D cell scaffold. Biomacromolecules.

[33] Lee SB (2004). Temperature/pH-sensitive comb-type graft hydrogels composed of chitosan and poly (N-isopropylacrylamide). J Appl Polym Sci.

[34] Lee J (2004). Synthesis and characterization of thermosensitive chitosan copolymer as a novel biomaterial. J Biomater Sci Polym Ed.

[35] Ha DI (2006). Preparation of thermo-responsive and injectable hydrogels based on hyaluronic acid and poly (N-isopropylacrylamide) and their drug release behaviors. Macromol Res.

[36] Wang J (2009). Cell adhesion and accelerated detachment on the surface of temperature-sensitive chitosan and poly (N-isopropylacrylamide) hydrogels. J Mater Sci Mater Med.

[37] Tan H (2009). Thermosensitive injectable hyaluronic acid hydrogel for adipose tissue engineering. Biomaterials.

[38] Gil ES, Hudson SM (2004). Stimuli-reponsive polymers and their bioconjugates. Prog Polym Sci.

[39] Ilmain F, Tanaka T, Kokufuta E (1991). Volume transition in a gel driven by hydrogen bonding. Nature.

[40] Tan R (2012). Thermo-sensitive alginate-based injectable hydrogel for tissue engineering. Carbohydr Polym.

[41] Delair T (2012). In situ forming polysaccharide-based 3D-hydrogels for cell delivery in regenerative medicine. Carbohydr Polym.

[42] Zhang Y (2014). Thermosensitive methyl cellulose-based injectable hydrogels for post-operation anti-adhesion. Carbohydr Polym.

[43] Peroglio M (2012). Injectable thermoreversible hyaluronan-based hydrogels for nucleus pulposus cell encapsulation. Eur Spine J.

[44] Abdi SIH (2012). In vivo study of a blended hydrogel composed of pluronic F-127-alginate-hyaluronic acid for its cell injection application. Tissue Eng Regen Med.

[45] Lee KY (2003). Hydrogel formation via cell crosslinking. Adv Mater.

[46] Koo LY (2002). Co-regulation of cell adhesion by nanoscale RGD organization and mechanical stimulus. J Cell Sci.

[47] Hu X, Gao C (2008). Photoinitiating polymerization to prepare biocompatible chitosan hydrogels. J Appl Polym Sci.

[48] Ifkovits JL, Burdick JA (2007). Photopolymerizable and degradable biomaterials for tissue engineering applications. Tissue Eng.

[49] Rice MA, Anseth KS (2004). Encapsulating chondrocytes in copolymer gels: bimodal degradation kinetics influence cell phenotype and extracellular matrix development. J Biomed Mater Res A.

[50] Bryant SJ (2004). Encapsulating chondrocytes in degrading PEG hydrogels with high modulus: engineering gel structural changes to facilitate cartilaginous tissue production. Biotechnol Bioeng.

[51] Peter SJ (2000). Marrow stromal osteoblast function on a poly (propylene fumarate)/β-tricalcium phosphate biodegradable orthopaedic composite. Biomaterials.

[52] Rouillard AD (2010). Methods for photocrosslinking alginate hydrogel scaffolds with high cell viability. Tissue Eng Part C Methods.

[53] Jeon O (2009). Photocrosslinked alginate hydrogels with tunable biodegradation rates and mechanical properties. Biomaterials.

[54] Jeon O (2010). Biodegradable, photocrosslinked alginate hydrogels with independently tailorable physical properties and cell adhesivity. Tissue Eng A.

[55] Burdick JA, Lovestead TM, Anseth KS (2003). Kinetic chain lengths in highly cross-linked networks formed by the photoinitiated polymerization of divinyl monomers: a gel permeation chromatography investigation. Biomacromolecules.

[56] Burdick JA (2005). Controlled degradation and mechanical behavior of photopolymerized hyaluronic acid networks. Biomacromolecules.

[57] Varghese S (2008). Chondroitin sulfate based niches for chondrogenic differentiation of mesenchymal stem cells. Matrix Biol.

[58] Garagorri N (2008). Keratocyte behavior in three-dimensional photopolymerizable poly (ethylene glycol) hydrogels. Acta Biomater.

[59] Kolb HC, Finn M, Sharpless KB (2001). Click chemistry: diverse chemical function from a few good reactions. Angew Chem Int Ed.

[60] Tan H, Rubin JP, Marra KG (2011). Direct synthesis of biodegradable polysaccharide derivative hydrogels through aqueous Diels-Alder chemistry. Macromol Rapid Commun.

[61] García-Astrain C, Avérous L (2018). Synthesis and evaluation of functional alginate hydrogels based on click chemistry for drug delivery applications. Carbohydr Polym.

[62] Hu W (2019). Advances in crosslinking strategies of biomedical hydrogels. Biomater Sci.

[63] Sarker B (2014). Fabrication of alginate–gelatin crosslinked hydrogel microcapsules and evaluation of the microstructure and physico-chemical properties. J Mater Chem B.

[64] Pettignano A (2017). Self-healing alginate–gelatin biohydrogels based on dynamic covalent chemistry: elucidation of key parameters. Mater Chem Front.

[65] Bleyer A (2008). The distinctive biology of cancer in adolescents and young adults. Nat Rev Cancer.

[66] Firooz A, Khatami A, Khamesipour A, Nassiri-Kashani M, Behnia F, Nilforoushzadeh M, Pazoki-Toroudi H, Dowlati Y. Intralesional injection of 2% zinc sulfate solution in the treatment of acute old world cutaneous leishmaniasis: a randomized, double-blind, controlled clinical trial. J Drugs Dermatol: JDD 4. 2005;4(1):73–9. 15696988

[67] Senapati S (2018). Controlled drug delivery vehicles for cancer treatment and their performance. Signal Transduct Target Ther.

[68] Soppimath KS (2001). Biodegradable polymeric nanoparticles as drug delivery devices. J Control Release.

[69] Su J (2011). Catechol polymers for pH-responsive, targeted drug delivery to cancer cells. J Am Chem Soc.

[70] Kumar S (2017). Controlled drug release through regulated biodegradation of poly (lactic acid) using inorganic salts. Int J Biol Macromol.

[71] Mo R, Jiang T, Gu Z (2014). Recent progress in multidrug delivery to cancer cells by liposomes. Nanomedicine.

[72] Dong Y (2014). Lipid-like nanomaterials for simultaneous gene expression and silencing in vivo. Adv Healthc Mater.

[73] Shih H, Lin C-C (2015). Photoclick hydrogels prepared from functionalized cyclodextrin and poly (ethylene glycol) for drug delivery and in situ cell encapsulation. Biomacromolecules.

[74] Li Y (2015). Biodegradable polymer nanogels for drug/nucleic acid delivery. Chem Rev.

[75] Gu FX (2007). Targeted nanoparticles for cancer therapy. Nano Today.

[76] Sun W, Gu Z (2015). Engineering DNA scaffolds for delivery of anticancer therapeutics. Biomater Sci.

[77] Maeda H (2000). Tumor vascular permeability and the EPR effect in macromolecular therapeutics: a review. J Control Release.

[78] Koo H (2011). In vivo targeted delivery of nanoparticles for theranosis. Acc Chem Res.

[79] Oh JK (2008). The development of microgels/nanogels for drug delivery applications. Prog Polym Sci.

[80] Kabanov AV, Vinogradov SV (2009). Nanogels as pharmaceutical carriers: finite networks of infinite capabilities. Angew Chem Int Ed.

[81] Kopeček J (2007). Hydrogel biomaterials: a smart future?. Biomaterials.

[82] Yallapu MM, Jaggi M, Chauhan SC (2011). Design and engineering of nanogels for cancer treatment. Drug Discov Today.

[83] Yan L (2012). Photo-cross-linked mPEG-poly (γ-cinnamyl-l-glutamate) micelles as stable drug carriers. Polym Chem.

[84] Park SY (2011). A smart polysaccharide/drug conjugate for photodynamic therapy. Angew Chem Int Ed.

[85] Beningo KA, Wang Y-l (2002). Fc-receptor-mediated phagocytosis is regulated by mechanical properties of the target. J Cell Sci.

[86] Hamidi M, Azadi A, Rafiei P (2008). Hydrogel nanoparticles in drug delivery. Adv Drug Deliv Rev.

[87] Vinogradov SV (2007). Polymeric nanogel formulations of nucleoside analogs. Expert Opin Drug Deliv.

[88] Abraham E (2017). Multifunctional cellulosic scaffolds from modified cellulose nanocrystals. ACS Appl Mater Interfaces.

[89] Danafar H (2014). Biodegradable m-PEG/PCL core-shell micelles: preparation and characterization as a sustained release formulation for curcumin. Adv Pharm Bull.

[90] Kamata H (2015). Design of hydrogels for biomedical applications. Adv Healthc Mater.

[91] Sudhakar Y, Kuotsu K, Bandyopadhyay AK (2006). Buccal bioadhesive drug delivery--a promising option for orally less efficient drugs. J Control Release.

[92] Shen H (2018). Chitosan–alginate BSA-gel-capsules for local chemotherapy against drug-resistant breast cancer. Drug Des Devel Ther.

[93] Glavas-Dodov M (2013). Wheat germ agglutinin-functionalised crosslinked polyelectrolyte microparticles for local colon delivery of 5-FU: in vitro efficacy and in vivo gastrointestinal distribution. J Microencapsul.

[94] Liu M (2017). Injectable thermoresponsive hydrogel formed by alginate-g-poly (N-isopropylacrylamide) that releases doxorubicin-encapsulated micelles as a smart drug delivery system. ACS Appl Mater Interfaces.

[95] Hosseinifar T (2018). Pressure responsive nanogel base on alginate-cyclodextrin with enhanced apoptosis mechanism for colon cancer delivery. J Biomed Mater Res A.

[96] Song W (2018). Magnetic alginate/chitosan nanoparticles for targeted delivery of curcumin into human breast cancer cells. Nanomaterials.

[97] Shad Pooneh Movahedi, Karizi Shohreh Zare, Javan Raheleh Safaie, Mirzaie Amir, Noorbazargan Hassan, Akbarzadeh Iman, Rezaie Hossein (2020). Folate conjugated hyaluronic acid coated alginate nanogels encapsulated oxaliplatin enhance antitumor and apoptosis efficacy on colorectal cancer cells (HT29 cell line). Toxicology in Vitro.

[98] Sun Z (2017). Bio-responsive alginate-keratin composite nanogels with enhanced drug loading efficiency for cancer therapy. Carbohydr Polym.

[99] Mirrahimi M (2019). A thermo-responsive alginate nanogel platform co-loaded with gold nanoparticles and cisplatin for combined cancer chemo-photothermal therapy. Pharmacol Res.

[100] Shtenberg Y (2018). Mucoadhesive alginate pastes with embedded liposomes for local oral drug delivery. Int J Biol Macromol.

[101] Matai I, Gopinath P (2016). Chemically cross-linked hybrid nanogels of alginate and PAMAM dendrimers as efficient anticancer drug delivery vehicles. ACS Biomater Sci Eng.

[102] Chen Z (2014). Non-small-cell lung cancers: a heterogeneous set of diseases. Nat Rev Cancer.

[103] Ahmad Z (2006). Alginate nanoparticles as antituberculosis drug carriers: formulation development, pharmacokinetics and therapeutic potential. Indian J Chest Dis Allied Sci.

[104] Society, A.C (2019). American cancer society.

[105] Siegel RL, Miller KD, Jemal A (2015). Cancer statistics, 2015. CA Cancer J Clin.

[106] Siegel RL, Miller KD, Jemal A (2016). Cancer statistics, 2016. CA Cancer J Clin.

[107] Fenn SL (2016). Dual-cross-linked methacrylated alginate sub-microspheres for intracellular chemotherapeutic delivery. ACS Appl Mater Interfaces.

[108] Quinn K (1985). Principles of burn dressings. Biomaterials.

[109] Coviello T (2007). Polysaccharide hydrogels for modified release formulations. J Control Release.

[110] Hong H-J (2008). Accelerated wound healing by smad3 antisense oligonucleotides-impregnated chitosan/alginate polyelectrolyte complex. Biomaterials.

[111] Sikareepaisan P, Ruktanonchai U, Supaphol P (2011). Preparation and characterization of asiaticoside-loaded alginate films and their potential for use as effectual wound dressings. Carbohydr Polym.

[112] Yin M (2015). Incorporation of magnesium ions into photo-crosslinked alginate hydrogel enhanced cell adhesion ability. J Tissue Eng Regen Med.

[113] Balakrishnan B (2005). Evaluation of an in situ forming hydrogel wound dressing based on oxidized alginate and gelatin. Biomaterials.

[114] Kamoun EA, Kenawy E-RS, Chen X (2017). A review on polymeric hydrogel membranes for wound dressing applications: PVA-based hydrogel dressings. J Adv Res.

[115] Vowden K, Vowden P (2017). Wound dressings: principles and practice. Surgery (Oxford).

[116] Saarai A (2012). On the characterization of sodium alginate/gelatine-based hydrogels for wound dressing. J Appl Polym Sci.

[117] Summa M (2018). A biocompatible sodium alginate/povidone iodine film enhances wound healing. Eur J Pharm Biopharm.

[118] Mohandas A (2015). Exploration of alginate hydrogel/nano zinc oxide composite bandages for infected wounds. Int J Nanomedicine.

[119] Pazoki-Toroudi H, Nilforoushzadeh MA, Ajami M, Jaffary F, Aboutaleb N, Nassiri-Kashani M, Firooz A. Combination of azelaic acid 5% and clindamycin 2% for the treatment of acne vulgaris. Cutan Ocul Toxicol. 2010;30(4);286–91. 10.3109/15569527.2011.581257 21612319

[120] Yu W (2016). Design of a novel wound dressing consisting of alginate hydrogel and simvastatin-incorporated mesoporous hydroxyapatite microspheres for cutaneous wound healing. RSC Adv.

[121] Singh R, Singh D (2012). Radiation synthesis of PVP/alginate hydrogel containing nanosilver as wound dressing. J Mater Sci Mater Med.

[122] Nazeri S (2015). Evaluation of effectiveness of honey-based alginate hyrogel on wound healing in a mouse model of rat. J Appl Biotechnol Rep.

[123] Straccia M (2015). Alginate hydrogels coated with chitosan for wound dressing. Mar Drugs.

[124] Hong N (2018). 3D bioprinting and its in vivo applications. J Biomed Mater Res B Appl Biomater.

[125] Ong CS (2018). 3D bioprinting using stem cells. Pediatr Res.

[126] Axpe E, Oyen M (2016). Applications of alginate-based bioinks in 3D bioprinting. Int J Mol Sci.

[127] Liu Q (2018). Preparation and properties of 3D printed alginate–chitosan Polyion complex hydrogels for tissue engineering. Polymers.

[128] Müller M (2017). Alginate sulfate–nanocellulose bioinks for cartilage bioprinting applications. Ann Biomed Eng.

[129] Faramarzi N (2018). Patient-specific bioinks for 3D bioprinting of tissue engineering scaffolds. Adv Healthc Mater.

