# Correction to: Alginate-based hydrogels as drug delivery vehicles in cancer treatment and their applications in wound dressing and 3D bioprinting

**DOI:** 10.1186/s13036-020-00239-0

**Published:** 2020-06-12

**Authors:** Farhad Abasalizadeh, Sevil Vaghefi Moghaddam, Effat Alizadeh, Elahe Akbari, Elmira Kashani, Seyyed Mohammad Bagher Fazljou, Mohammadali Torbati, Abolfazl Akbarzadeh

**Affiliations:** 1 Department of Traditional Medicine, Faculty of Traditional Medicine, Tabriz University of Medical Sciences, Tabriz, Iran; 2 Drug Applied Research Center, Tabriz University of Medical Sciences, Tabriz, Iran; 3 Department of Medical Biotechnology, Faculty of Advanced Medical Sciences, Tabriz University of Medical Sciences, Tabriz, Iran; 4 Higher Education Institute of Rab-Rashid, Tabriz, Iran; 5 Department of Medical Nanotechnology, Faculty of Advanced Medical Sciences, Tabriz University of Medical Sciences, Tabriz, Iran; 6 Department of Food Science and Technology, Faculty of Nutrition, Tabriz University of Medical Sciences, Tabriz, Iran; 7 Tuberculosis and Lung Disease Research Center of Tabriz, Tabriz University of Medical Sciences, Tabriz, 22 5154853431 Iran; 8 Universal Scientific Education and Research Network (USERN), Tabriz, Iran

**Correction to: Journal of Biological Engineering (2020) 14:8**

**https://doi.org/10.1186/s13036-020-0227-7**

Following the publication of this article [1] the authors informed us of the following errors:

1. Figure 18 should be removed, since this is the same as Fig. 16.
2. Figure 17 legend should be replaced by Fig. 18 legend to read:
  Fig. 17 Patient-Specific Platelet-Rich Plasma (PRP) bioink using 3D bioprinting of alginate scaffold a Schematic of PRP extraction and its incorporation with alginate to form patient-specific bioink b Schematic of proposed bioprinting process c PRP incorporated alginate scaffold containing fluorescence particles. d Images of different PRP-alginate constructs. In the production of these constructs 0.04% (w/v) CaCl2, 50 U ml − 1 PRP, and 1% (w/v) alginate was used. e, f The fabricated constructs could easily be removed from the substrate without losing their integrity. g Metabolic activity of mesenchymal stem cells (MSCs) treated with alginate and alginate/PRP over 5 days without any growth factor. h Metabolic activity of human umbilical vein endothelial cells (HUVECs) treated with alginate and alginate/PRP over 3 days without any growth factor. (*P < 0.05; **P < 0.01, ***P < 0.001) [129]. Faramarzi, N., et al., Patient-Specific Bioinks for 3D Bioprinting of Tissue Engineering Scaffolds. Advanced healthcare materials, 2018. 7(11), Copyright (2020)

It is also clarified here that Figs. 9, 10, 11, 12, 13 and 15 were reproduced with permission from the copyright holders.
